# Smoothing a rugged protein folding landscape by sequence-based redesign

**DOI:** 10.1038/srep33958

**Published:** 2016-09-26

**Authors:** Benjamin T. Porebski, Shani Keleher, Jeffrey J. Hollins, Adrian A. Nickson, Emilia M. Marijanovic, Natalie A. Borg, Mauricio G. S. Costa, Mary A. Pearce, Weiwen Dai, Liguang Zhu, James A. Irving, David E. Hoke, Itamar Kass, James C. Whisstock, Stephen P. Bottomley, Geoffrey I. Webb, Sheena McGowan, Ashley M. Buckle

**Affiliations:** 1 Biomedicine Discovery Institute, Department of Biochemistry and Molecular Biology, Monash University, Clayton, Victoria 3800, Australia; 2 Medical Research Council Laboratory of Molecular Biology, Francis Crick Avenue, Cambridge, CB2 0QH, United Kingdom; 3 Department of Chemistry, University of Cambridge, Lensfield Road, Cambridge, CB2 1EW, United Kingdom; 4 Programa de Computação Científica, Fundação Oswaldo Cruz, 21949900 Rio de Janeiro, Brazil; 5 Faculty of Information Technology, Monash University, Clayton, Victoria 3800, Australia; 6 Wolfson Institute for Biomedical Research, University College London, Gower Street, London, WC1E 6BT, United Kingdom; 7 ARC Centre of Excellence in Advanced Molecular Imaging, Monash University, Clayton, Victoria 3800, Australia; 8 Biomedicine Discovery Institute, Department of Microbiology, Monash University, Clayton, Victoria 3800, Australia

## Abstract

The rugged folding landscapes of functional proteins puts them at risk of misfolding and aggregation. Serine protease inhibitors, or serpins, are paradigms for this delicate balance between function and misfolding. Serpins exist in a metastable state that undergoes a major conformational change in order to inhibit proteases. However, conformational labiality of the native serpin fold renders them susceptible to misfolding, which underlies misfolding diseases such as α_1_-antitrypsin deficiency. To investigate how serpins balance function and folding, we used consensus design to create *conserpin,* a synthetic serpin that folds reversibly, is functional, thermostable, and polymerization resistant. Characterization of its structure, folding and dynamics suggest that consensus design has remodeled the folding landscape to reconcile competing requirements for stability and function. This approach may offer general benefits for engineering functional proteins that have risky folding landscapes, including the removal of aggregation-prone intermediates, and modifying scaffolds for use as protein therapeutics.

The rugged energy landscapes of functional proteins reflect the delicate balance between efficient folding and function^1^,^2^. For proteins to fold, the interactions of the native state must outweigh the non-native interactions, which result in a funnel-shaped energy landscape^3^,^4^,^5^. However, it is not obvious how the myriad of non-covalent interactions that stabilise the native state can do so selectively over the vastly larger number of non-native conformations. Effective protein engineering has typically focused on stabilising low energy configurations as observed in X-ray crystallography or nuclear magnetic resonance (NMR) spectroscopy^6^,^7^,^8^,^9^. However, engineering robust proteins with funnel shaped energy landscapes may require not only stabilisation of the native state (positive design)^10^,^11^,^12^, but also destabilisation of non-native states (negative design)^12^,^13^,^14^,^15^. This is especially true for engineering proteins with complex and rugged folding pathways, which often exhibit a delicate balance between function and misfolding^1^,^2^.

Such a balance is exemplified by members of the serine protease inhibitor, or serpin superfamily^16^,^17^,^18^,^19^. Inhibitory members fold to a metastable native state that undergoes a major conformational change in order to inhibit target proteases^20^. The inhibitory mechanism of serpins is structurally well understood^20^. Briefly, a target protease initially interacts with and cleaves the serpin reactive center loop (RCL) that protrudes from the main body of the molecule. Following RCL cleavage, but prior to the final hydrolysis of the acyl enzyme intermediate, the RCL inserts into the central β-sheet to form an extra strand^20^,^21^. Since the protease is still covalently linked to the serpin, the process of RCL insertion results in the translocation of the protease to the opposite end of the molecule. In the final complex, the protease active site is distorted and trapped as the acyl enzyme intermediate^20^,^22^. This remarkable conformational change is termed the stressed [S] to relaxed [R] transition and is accompanied by a major increase in stability of the serpin protein.

As a consequence of folding to a metastable active state, serpins are prone to misfolding. Without being cleaved by a protease, the serpin RCL can self-insert, either partially (delta), or fully (latent)^16^; or polymerize by insertion of the RCL of one serpin into the body of another serpin^23^,^24^,^25^. Both such RCL insertion events result in a more stable protein species that is no longer functional as a protease inhibitor. Misfolding of the archetypal serpin, α1-antitrypsin (α1-AT), results in a deficiency of active protein, inducing emphysema through uncontrolled protease activity, and the retention of α1-AT polymers in the liver that induce cell death^18^,^26^. Serpin misfolding and serpinopathies are a direct result of the ‘risky’ energy landscape required to fold the protein to a metastable state^1^,^27^. To investigate how the folding energy landscape of serpins balances the competing requirements for function and stability we used consensus design to build a synthetic serpin. Consensus design is based on the hypothesis that at a given position in a multiple sequence alignment (MSA) of homologous proteins, the respective consensus amino acid contributes more than average to the stability of the protein than non-consensus amino acids^28^,^29^,^30^,^31^. The efficacy of consensus design has been demonstrated to increase the stabilities of a wide range of proteins, usually by stabilising the native state^29^,^31^,^32^,^33^,^34^,^35^,^36^,^37^,^38^. However, its potential for altering folding landscapes has not been thoroughly explored^29^,^31^,^32^,^33^,^34^,^35^,^36^,^37^,^38^.

Hypothesizing that a serpin reflecting a highly conserved sequence may offer insight into the delicate balance between folding and function, we designed *conserpin (con*sensus *serpin*). Characterization of its function, structure and folding reveal a serpin that is inhibitory, folds reversibly, is thermostable and resistant to polymerisation. Our results suggest that consensus design has smoothed the folding landscape, reducing the lifetime of aggregation-prone intermediates. This work provides insights into the serpin function-stability balance and emphasises the wider potential for consensus design to remodel the risky folding landscapes of functional proteins.

## Results

### Conserpin is an inhibitory serpin

To design *conserpin* we used the consensus approach and a previously reported MSA of 219 serpin sequences^39^. Conserpin (396 aa) shares the highest similarity with α1-AT (137 residue differences; 62% sequence identity). There is an overall loss of 10 residues located at the N-terminus of the D-helix and C-terminus of the protein. The RCL contains 7 residue differences compared to α1-AT, notably an arginine at P1 compared to the methionine of α1-AT, and the deletion of a residue at P2.

**Figure 1:**
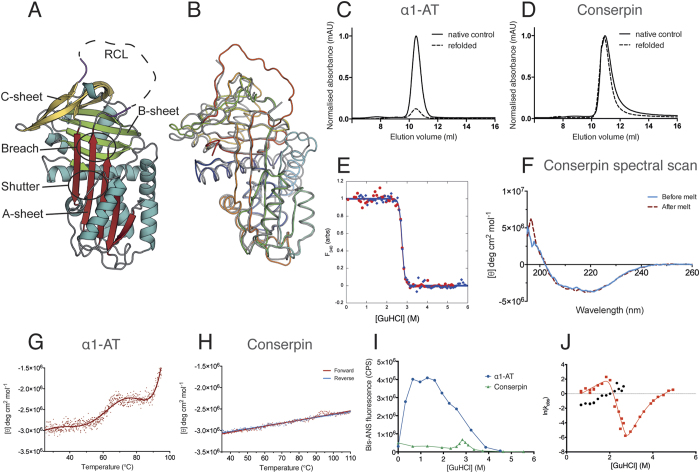
Conserpin conforms to the serpin fold and has superior biophysical properties compared with α1-AT. **(A)** Cartoon representation of the 2.4 Å X-ray crystal structure of native conserpin, identifying the breach and shutter regions, the A, B and C sheets (colored in red, green and yellow respectively), and the RCL stumps (magenta). **(B)** Structural alignment of conserpin (grey) with α1-AT (PDB: 3NE4; spectrum, blue to red). Root mean square deviation (RMSD) = 0.91 Å across 296 backbone Cα atoms. Chemical refolding of **(C)** α1-AT and **(D)** conserpin shows that conserpin can refold to a monomer. Chromatograms from a Superdex 75 10/300 size exclusion column are shown. Final protein concentrations loaded onto column were 2 μM. Samples were unfolded in 5 M GuHCl and then diluted out to 0.5 M GuHCl (dotted line). Control samples of native protein are shown as the solid black line. **(E)** Intrinsic fluorescence equilibrium unfolding (red dots) and refolding (blue diamonds) curves of conserpin coincide, demonstrating reversible folding. **(F)** CD spectral scans of conserpin before (solid blue line) and after (dashed red line) heating to 110 °C. Variable temperature thermal melts of **(G)** α1-AT and **(H)** conserpin as measured by CD at 222 nm. **(I)** Conserpin shows a significant reduction of intermediate formation during bis-ANS fluorescent equilibrium unfolding of α1-AT (blue circles) and conserpin (green triangles). **(J)** Kinetic unfolding and refolding experiments. The plot shows the [GuHCl]-dependence of the natural logarithm of the rate constants for unfolding and refolding of conserpin (chevron plot). Two discernable refolding rates are observed (red squares, fast rate; black circles, slower folding rate). The positive slope in each refolding arm suggests the presence of intermediate species that have to partially unfold to reach the native state.

Purified conserpin inhibits trypsin with a stoichiometry of inhibition (SI) of 1.8 and a *k*_ass_^app^ of 7.5 × 10^6 ^M^−1^ s^−1^ and hence a rate of association (*k*_ass_) of 1.4 × 10^7 ^M^−1^ s^−1^ (Fig. S1A–C). Higher order complex formation of conserpin with trypsin was observed on SDS PAGE; however, it was atypical compared to α1-AT (Fig. S1D). This unusual behaviour and the increased SI of conserpin may be a consequence of shortening the RCL on the ‘prime’ side of the recognition sequence for trypsin, or due to other biophysical differences. The crystal structure of conserpin (Table S1), confirms that it adopts the archetypal native serpin fold (Fig. 1A,B). Taken together, we propose that inhibition by conserpin occurs via the classical serpin mechanism.

### Conserpin folds reversibly, is thermostable and resistant to polymerization

The majority of serpins unfold through an aggregation-prone intermediate ensemble and do not completely refold after chemical and/or thermal denaturation^40^,^41^,^42^,^43^,^44^,^45^,^46^,^47^. This is exemplified by α1-AT, which shows a very small amount of refolded monomer via chemical denaturation, rapid dilution and gel filtration (Fig. 1C). In contrast, conserpin refolds to a monomeric state (Fig. 1D). Equilibrium chemical unfolding and refolding curves overlay well, revealing a midpoint of denaturation, [D]_50_, of 2.75 ± 0.10 M, an equilibrium *m*-value, *m*_D-N_, of 8.45 ± 0.65 kcal mol^−1^ M^−1^, and hence a stability, ∆*G*_D-N_, of −23.2 ± 2.0 kcal mol^−1^ (Fig. 1E). The correlation of unfolding and refolding curves, the single unfolding transition, and the steep *m*-value all suggest minimal formation of an intermediate ensemble. Refolded conserpin retained inhibitory activity, resulting in no significant change in SI (increased from 1.8 to 2.3, Fig. S1E), confirming that conserpin refolds to the native state after chemical denaturation.

Variable temperature circular dichroism (CD) thermal melt analysis at 222 nm reveals a highly thermostable protein with no defined unfolding transition up to a temperature of 110 °C (Fig. 1H). Far-UV spectral scans before and after the thermal melt showed no change in signal, indicating no detectable heat-induced structural changes (Fig. 1F). This contrasts with α1-AT, which upon heating undergoes a three-state transition with an initial midpoint temperature (*T*_*m*_) of 61.8 °C and an incomplete transition that starts at 90 °C (consistent with other reports^48^,^49^; Fig. 1G). Upon cooling of α1-AT, we observed a white precipitate in the cuvette, consistent with irreversible aggregation. Refolding transverse urea gradient (TUG) gels further demonstrate that conserpin is more resistant to polymerization than α1-AT, which mostly formed polymers on refolding, with no formation of native protein (Fig. S2).

To test if conserpin undergoes a transition to the more stable latent state upon heating, we assessed its inhibitory activity and structure after heating at 80 °C for 20 minutes. Heating caused a complete loss in inhibitory activity (Fig. S1F), suggesting formation of the latent state, which was then confirmed by native PAGE (Fig. S3A) and crystal structure determination (Table S1 & Fig. S3B).

### Conserpin avoids polymerization by minimizing formation of folding intermediates

Although equilibrium unfolding/refolding data using intrinsic fluorescence indicated minimal formation of intermediates in the folding pathway of conserpin (Fig. 1E), this method is dependent on the difference in solvation of tryptophan residues during unfolding/refolding. In order to more thoroughly interrogate folding intermediates, we repeated the equilibrium unfolding experiments in the presence of bis-ANS (Fig. 1I). In native conditions, both folded conserpin and α1-AT show similar levels of fluorescence, however, by ~ 1 M GuHCl, a high intensity fluorescent peak indicated the presence of a folding intermediate(s) for α1-AT (Fig. 1I), consistent with previous reports^47^. In contrast, the unfolding profile of conserpin in bis-ANS shows a small, sharp peak at approximately 3 M GuHCl (Fig. 1I). This is consistent with our [D]_50_ measurement by intrinsic fluorescence (Fig. 1E) and confirms our hypothesis that conserpin has reduced intermediate ensemble formation.

To observe the kinetics of the folding intermediate, we used rapid mixing techniques during unfolding and refolding (Fig. 1J). As expected, the unfolding traces fitted well to a single exponential. When the protein was refolded from an equilibrated denatured solution (single-jump), the resulting traces could not be fitted to fewer than three exponentials (Fig. S1G) and showed inconsistencies between repeats (likely due to aggregate from previous runs). However, unfolding native conserpin followed by refolding (double-jump) resulted in more consistent refolding traces that fitted to a double exponential (SI methods; Fig. S1H). Both single and double jump refolding identified two rates that were independent of the delay time (Figs S1I,J and 1J). Plausible explanations for the presence of two refolding rates are: two denatured states folding on different timescales (e.g. folding limited by proline isomerisation); a fast rate of refolding to an intermediate, followed by a slow rate of refolding from that intermediate; or two fluorophores reporting on independent folding events (e.g. two independently nucleating subdomains). In our data, it is most likely that we are detecting folding from two similarly structured ground states. If we were observing a fast rate, followed by a slow rate, we should expect the fast rate to become kinetically invisible when the two rates cross (~2 M GuHCl), which it does not. Similarly, if there are two independent folding events, then the relative amplitudes of each rate should be consistent, which they are not. Most interestingly, the refolding *m-values* are positive at low concentrations of denaturant (<2 M), suggesting that the two populated ground states are more structured than the subsequent folding transition state(s). Therefore, the starting states cannot be denatured states, and must be structured intermediates (I_1_ and I_2_) that fold on different time-scales (Figs 1J and S1I,J). The fast folding rate (red squares) matches up with the unfolding rate at the expected [D]_50%,_ (2.75 M), verifying that this rate shows folding over the major transition state. The “rollover” in this rate demonstrates that the first intermediate I_1_ is in rapid pre-equilibrium with the denatured state (D) and there is a switch in ground state from I_1_ to D when the two species are of equal stability (2 M GuHCl, red squares in Fig. 1J). The second intermediate I_2_ (Fig. 1J, black circles) shows an almost identical folding *m*-value and, assuming this also folds over the major transition state, is likely to be very similar in structure to I_1_. However, I_2_ is more stable than I_1_ and persists until the denaturant midpoint (2.75 M). As such, we propose that I_1_ is likely to be the previously observed polymerogenic folding branch point^19^,^40^,^47^,^50^,^51^,^52^,^53^. As this species is highly aggregation prone in other serpins, it is possible that the second intermediate (I_2_) is a multimer of the first intermediate.

### Global structural features of native conserpin are not typical for a thermostable protein

**Figure 2:**
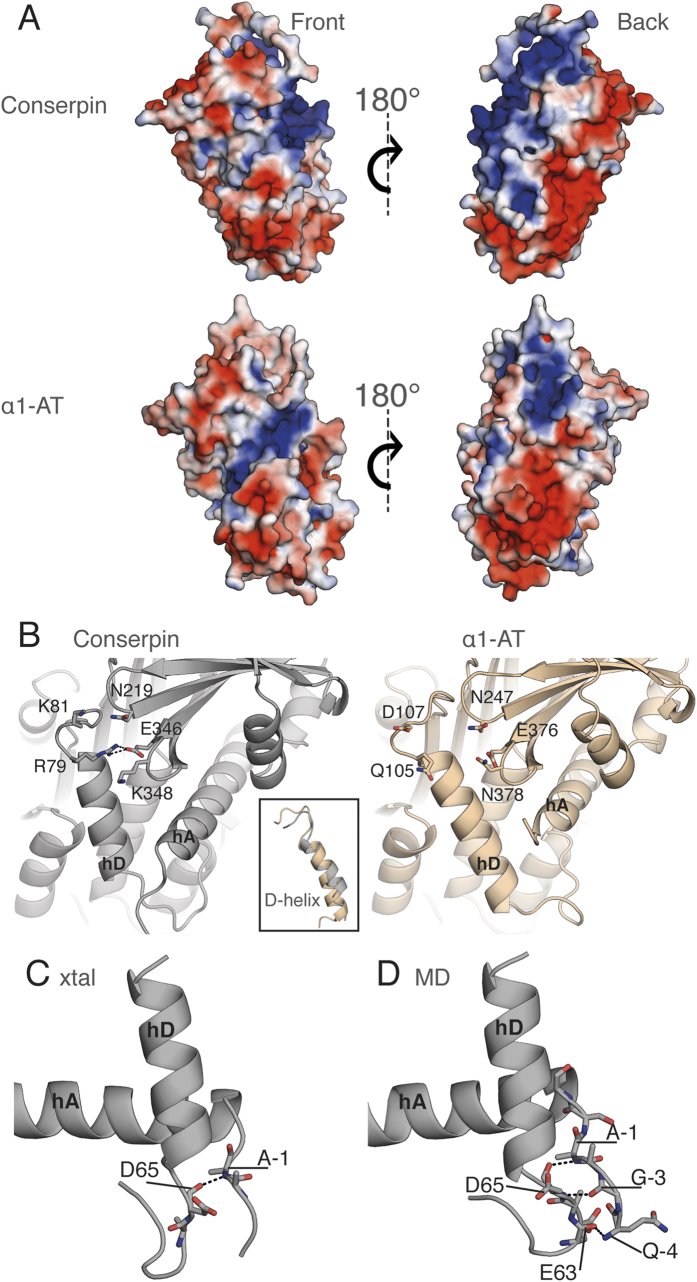
Structural analysis reveals alterations of the electrostatic surface and stabilization of the D-helix in conserpin. **(A)** The electrostatic potential surface of conserpin and α1-AT models (blue =  +ve, red = −ve), in the same orientation as Fig. 1A (front) and a 180° rotation reveals an overall increase in positive charge on the back face of conserpin. **(B)** The introduced salt bridge in hD of conserpin with residues Q105R_79_ and E376_346_. There is no comparable interaction present in α1-AT. Inset shows the shortened D-helix in conserpin. **(C)** H-bonding between A-1 of the extended N-terminus and D65 of hD, as seen in the conserpin crystal structure. **(D)** Persistent hydrogen bonding between Q-4, G-3 and A-1 of the extended N-terminus and E63 and D65 of hD in conserpin as seen in MD simulation.

Our data imply that the folding reversibility and low polymerization propensity of conserpin is due to alteration of the folding landscape, resulting in minimal formation of a folding intermediate. Comparison of native and latent state conserpin structures with available native, latent and cleaved structures of α1-AT^54^,^55^,^56^, plasminogen activator inhibitor 1 (PAI-1)^57^,^58^, α1-antichymotrypsin (ACH)^59^, neuroserpin^60^,^61^, antithrombin^62^ and the thermostable serpins, thermopin^63^ and tengpin^64^ reveal that despite having the highest thermostability, native conserpin has the fewest H-bonds and salt bridges (Table S2). Further, native state conserpin has the largest accessible surface area and largest solvent inaccessible cavity volume of all assessed serpins. These characteristics are unusual for thermostable proteins, which typically feature more interactions and optimized packing compared to their mesophilic counterparts^37^,^65^,^66^,^67^,^68^,^69^,^70^,^71^,^72^,^73^. Comparison of the electrostatic surface potential of conserpin with that of α1-AT reveals minor differences on the surface-exposed face of the A-sheet, whilst the opposite face of the molecule is substantially more positively charged (Fig. 2A), consistent with the reported aggregation resistance of proteins featuring increased electrostatic surface potential^74^,^75^. In contrast to the majority of mesophilic proteins and their thermophilic homologues, a correlation between overall number of H-bonds/salt bridges and thermostability is not apparent for serpins, which must balance the relative stabilities of native and RCL-inserted states to enable unique conformational plasticity underpinning inhibitory function^63^,^64^ (Table S2). This reasoning suggests that more subtle, context-dependent structural and dynamical features play a more dominant role in conserpin, which we explore next.

### Favorable interactions and reduced dynamics surrounding the D-helix

Given the conformational plasticity required for serpin function, we next performed molecular dynamics (MD) simulations for 0.5 μs at 300 K in triplicate for both conserpin and α1-AT. Both systems reach equilibrium by 150 ns (Fig. S4A). Although the increased mobility of the RCL and the C-terminus of hA of conserpin leads to a higher overall RMSD, inspection of root mean square fluctuations (RMSFs) shows conserpin to exhibit an overall reduction in dynamics in the majority of regions, specifically the extended N-terminus of hA, hC/hD loop, hD, hE, hF, hG, hH (Fig. S4B,C). This is further supported by a large reduction in conformational sampling as shown by principle component analysis (Fig. S4D). The most notable reduction in dynamics is in the D-helix (hD; RMSD of 0.58 vs. 1.65 Å; Figs 2B and S4B,C). The D-helix of α1-AT has been implicated in stability; notably two mutations (T114F_α1-AT_ and G117F_α1-AT_) stabilize the D-helix and rescue the polymerogenic Z-variant^76^,^77^. The D-helix of conserpin is shortened by the deletion of five residues, four at the N-terminus (L84, E86, I87 and P88 in α1-AT) and one at the C-terminus (Q109 in α1-AT; Figs 2B & S5A). The deletion of L84_α1-AT_ and I87_α1-AT_ reduces overall hydrophobicity without affecting the packing of hD against the core of conserpin (Fig. S5A). Residue numbering will adhere to the following convention unless explicitly stated: Q105_α1-AT_ or R79_conserpin_ or Q105R_79_, where Q105 from α1-AT has been mutated to an R, which is residue number 79 in conserpin.

The rigidity of hD in conserpin is probably due to a salt bridge between Q105R_79_ of hD and E376_346_ and interactions of the N-terminus with hD. The salt bridge between the B-sheet and hD is present throughout the MD simulation and possibly stabilizes the top of the D-helix (Fig. 2B). In contrast, there are no similar salt bridges in the α1-AT crystal structure or during MD (Fig. 2B). Rather, hD in α1-AT undergoes conformational rearrangement and loss of secondary structure in one of the replicates (Fig. S5B). This is consistent with other reports, which indicates that minor changes to hD may accelerate or reduce polymer formation^76^,^77^. The N-terminus in conserpin is extended by the addition of a purification tag. Four residues of the extension were resolved in the crystal structure and a single H-bond is observed between the backbone of residue A-1_conserpin_ and the N-terminus of hD (D65_conserpin_; Fig. 2C). This H-bond is persistent throughout MD and extends to form a small β-sheet (Fig. 2D). Therefore, the extended N-terminus may impart stability to hD and may reflect similar interactions seen in the naturally extended N-termini of thermophilic serpins^63^,^64^. Taken together, our observations suggest that optimized interactions in and around hD increase the stability of the native state.

### The electrostatic network of the serpin breach region is extended in conserpin

**Figure 3:**
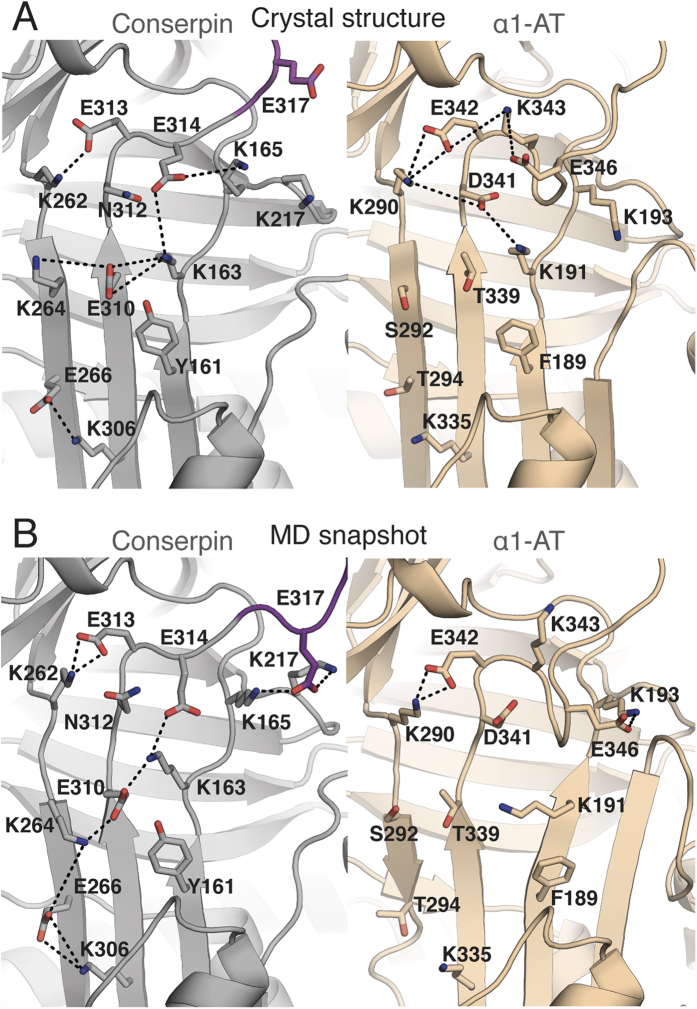
The electrostatic network of the breach region is extended in conserpin. **(A)** A-sheet salt bridge interactions (dashed lines) in the crystal structures of conserpin (carbon atoms in grey) and α1-AT (carbon atoms in wheat; PDB: 3NE4). **(B)** A simulation snapshot taken at 500 ns, showing A-sheet salt bridge interactions as described above. The modeled RCL of conserpin is colored magenta.

The breach region, consisting of a highly conserved electrostatic network between residues E342_α1-AT_, K290_α1-AT_ and D341_α1-AT_ at the top of the A-sheet is important for controlling the conformational change that drives protease inhibition^39^,^49^,^79^. This network is significantly extended in conserpin, compared to α1-AT (Fig. 3A). Specifically, the mutations of T339E_310_ and S292K_264_ contribute to a salt bridge network spanning s3A, s5A and s6A with K191_163_. T294E_266_ also forms a new salt bridge with K335_306_ between s6A and s5A, whilst D341N_312_ mediates an unfavorably charged cluster of E310_conserpin_, E313_conserpin_ and E314_conserpin_ that is not present in α1-AT (Fig. 3A). These observations are interesting in the context of serpin polymerization, which involves insertion of the RCL and/or s5A from one molecule into the flexible A-sheet of another^19^,^23^,^24^,^40^,^51^,^78^. In particular, the disease-causing Z-variant, E342K_α1-AT_ induces repulsion with K290_α1-AT_, which either retards the formation of the A-sheet during folding, increasing the lifetime of the polymerogenic intermediate ensemble, or destabilizes the structure and increases the dynamics of the native state, allowing for s5A and s6A to separate, and reduce the energy barrier for polymerization^19^,^40^,^80^,^81^,^82^.

It is difficult to ascertain the effect of the extended salt bridge network on A-sheet dynamics during folding of conserpin as MD simulations only describe the dynamics of the native state. Nevertheless, simulations of α1-AT reveal its A-sheet salt bridge network to be weaker over time (Fig. 3B), allowing for the transient separation of strands s5A and s3A (Fig. S6). In contrast, the extensive network in conserpin is present throughout the majority of the simulation, with additional interactions being formed, notably an alternate conformation of K264_conserpin_ mediating interactions between E310_conserpin_, and E266_conserpin_ (Fig. 3B). Furthermore, E317_conserpin_ in the RCL of conserpin is able to adopt a stable conformation, mediating the salt-bridge between K165_conserpin_ and K217_conserpin_, with K165_conserpin_ forming transient interactions to E314_conserpin_. Equilibrium and kinetic folding studies of α1-AT provide compelling evidence for the late folding of s5A during transition through the polymerogenic intermediate state^19^,^40^. Taken together, our observations suggest an improved energetically stable conserpin native state with possible increases to the folding cooperativity in this region, which may also be augmented by the hydrophobic core behind the A-sheet.

### Biophysical and structural analysis of Z-conserpin

Intrigued by the stabilizing electrostatic interactions in the breach region of conserpin, we assessed the effect of introducing the disease-causing Z-mutation, E342K_313_, into conserpin. Mutation of E342_α1-AT_ to a lysine results in an increased propensity of α1-AT to polymerize in the endoplasmic reticulum of hepatocytes, leading to a lack of secretion into the circulation^26^. Studying the effects of the Z-variant in α1-AT is difficult due to expression as insoluble aggregate^76^,^83^. The most likely mechanism of Z-variant polymerization involves perturbation of the folding energy landscape, thus increasing the lifetime of the polymerogenic intermediate ensemble^24^,^40^,^81^,^83^. Although there is evidence to suggest that the Z-mutation also results in structural and dynamic changes to the native state^80^,^82^,^84^, a recent crystal structure of Z α1-AT shows minimal perturbation in comparison to wild-type^83^. In order to investigate the effects of a highly destabilizing mutation on conserpin, we introduced E342K_313_ into conserpin to produce Z-conserpin.

Z-conserpin expressed well as a soluble monomer in *E. coli*, which has not been possible with Z α1-AT^76^,^83^. Z-conserpin showed a highly similar inhibitory profile to conserpin, with an SI of 2.3 and a *k*_ass_ of 2.1 × 10^7 ^M^−1^ s^−1^ (Fig. S7A,B). Z-conserpin exhibits reversible, two-state folding upon chemical denaturation (Fig. S7C,D). The equilibrium unfolding and refolding curves overlay almost perfectly, revealing a midpoint of denaturation, [D]_50_ to be 2.51 ± 0.01 M, an equilibrium *m*-value, *m*_D-N_, of 5.18 kcal mol^−1^ M^−1^, and a stability ∆G_D-N_, of −12.8 kcal mol^−1^ (a loss of −10.04 kcal mol^−1^; Fig. S7D). As with conserpin, equilibrium data did not reveal the presence of an intermediate species. We therefore repeated equilibrium unfolding using bis-ANS fluorescence, detecting a fluorescent peak at ~2.5 M GuHCl, that is slightly broader and more intense than observed in conserpin, indicating an increase in the intermediate ensemble population, but still smaller than in α1-AT (Fig. S7E). Variable-temperature far-UV CD melting curves in 2 M GuHCl gave a *T*_*m*_ of 60.7 °C (conserpin *T*_*m*_ = 72.5 °C; (Fig. S7F)). Native PAGE shows conserpin to remain monomeric except when heated to 90 °C for 10 minutes, whilst Z-conserpin has a complete loss of monomer at 80 °C and forms a slightly higher molecular weight species when heated to 70 °C for 10 minutes (Fig. S7G). The crystal structure of native Z-conserpin (Table S1) reveals almost no structural differences upon mutation (backbone RMSD = 0.23 Å); the sole differences surrounding E342K_313_ are small side-chain shifts of K342_313_ and K290_262_, most likely as a result of electrostatic repulsion (Fig. S7H). A caveat is one local residue difference, K343E_314_, in conserpin that may partially negate the effects of E342K_313_, due to its salt bridge with K165_conserpin_. As such, future studies of the double mutant E342K_313_/E314K_conserpin_ would be insightful. Regardless, the structure of Z-conserpin reveals essentially no structural changes to the native state which disagrees with reports of structural perturbations within the native state^80^,^82^,^84^; therefore favoring the mechanism of Z-variant polymerization via a folding intermediate^83^. However, the intermediate versus native state polymerization mechanisms may be reconciled if the intermediate ensemble is native-like in structure, consistent with our kinetic (un)folding data for conserpin. Considering the evidence in support of this for a wide range of proteins^85^, our data is therefore consistent with the Z-mutation altering the folding energy landscape, possibly by lowering the kinetic barrier of the unfolding transition to the polymerogenic intermediate ensemble^81^,^83^.

### Importance of A-sheet/F-helix hydrophobic core packing

The hydrophobic core buried by the A-sheet is important for serpin stability^48^,^86^,^87^. Amongst 19 mutations designed to probe the stability of α1-AT, seven mutations in the hydrophobic core were found to be stabilizing^86^. Four of these mutations are found in conserpin (T59S_37_, T68A_46_, A70G_48_ and M374I_244_). In the remaining three mutations, the local environment adapts to improve packing and local interactions (Fig. S8).

**Figure 4:**
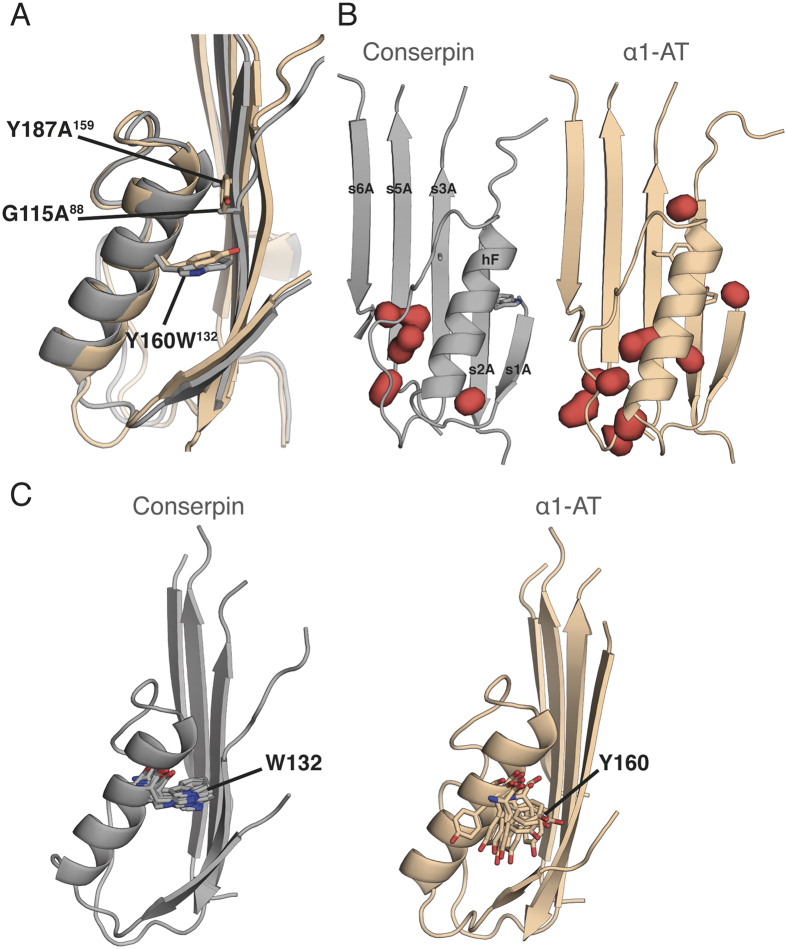
W160 stabilizes hF in conserpin. **(A)** A structural overlay of hF in conserpin (grey) and α1-AT (wheat), highlighting the positions of Y160W_132_, Y187A_159_ and G115A_88_**. (B)** Solvent inaccessible cavities (red blobs) surrounding hF of conserpin and α1-AT. Y160W_132_ reduces cavity volumes from 233.8 to 120.9 Å^3^. **(C)** MD simulation frames (every 50 ns), highlighting the dynamic differences of W132 in conserpin and Y160 in α1-AT.

Packing between hF and the A-sheet also stabilizes the native serpin state, with hF acting as a physical barrier for RCL insertion into the A-sheet during protease inhibition and polymerization^19^,^88^,^89^,^90^,^91^. Conserpin contains three mutations in this region (Fig. 4A); Y187A_159_ and G115A^88^, which allow s2A to more tightly pack against hF, and Y160W_132_, which further improves the packing density (Fig. 4B). This is consistent with mutagenesis studies of α1-AT, where Y160A resulted in a 5 °C decrease in *T*_*m*_ and was attributed to the loss of a hydrogen bond and formation of a cavity^88^. In contrast, Y160W raised the *T*_*m*_ of α1-AT to 65 °C, and slowed the rate of polymerization^88^. MD reveals hF of conserpin to be slightly less flexible than that of α1-AT, with W160_132_ remaining conformationally locked compared to Y160 of α1-AT, which frequently flips in and out of the hydrophobic pocket (Fig. 4C). Interactions within the “clasp” motif at the F-helix are structurally conserved in conserpin and maintained throughout simulation, consistent with its proposed role in regulating conformational change^92^. Taken together, these changes likely contribute to the stability of the native state.

### Remodeling the B/C barrel, a folding nucleus

**Figure 5:**
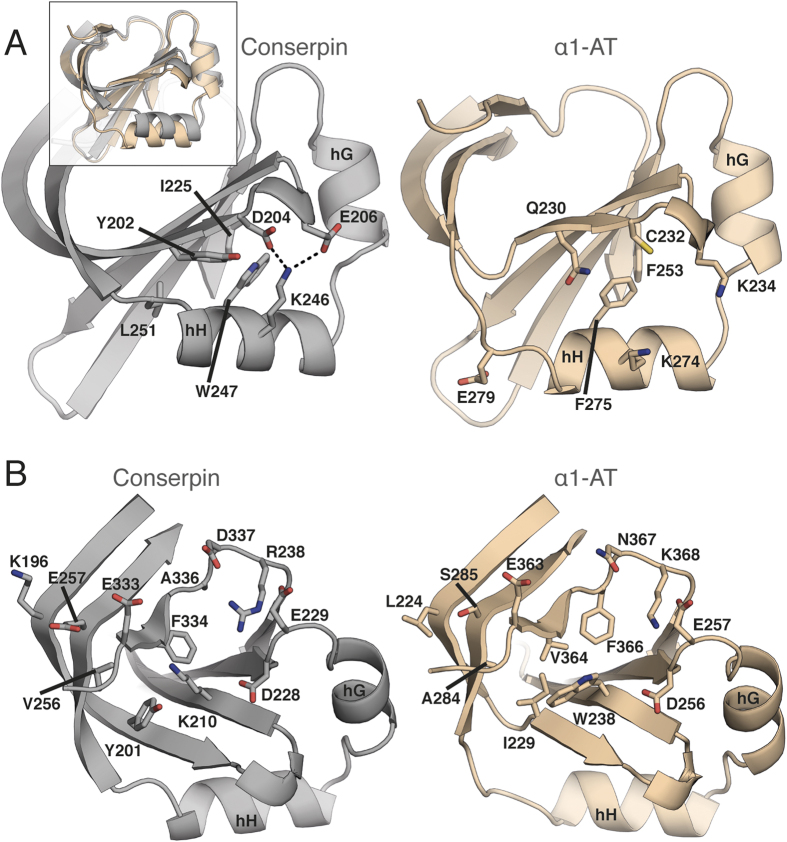
Structural analysis of the B/C barrel in conserpin (grey) and α1-AT (wheat). **(A)** Stabilizing hydrophobic mutations surrounding F275W_247_. **(B)** Remodeling of the inner barrel surrounding W238K_210_.

Formation of the B/C barrel is thought to occur early in the folding pathway of α1-AT, preceding formation of the A-sheet and acting as a “kinetic trap” that captures the RCL and prevents folding to other more stable states^19^,^22^,^40^,^55^,^93^. Conserpin contains several mutations in the B/C barrel that improve hydrophobic packing and form favorable interactions within the native state (Fig. 5A). Specifically, F275W_247_ and E279L_251_ allow tighter packing of hH. The introduction of a small salt-bridge network between K274_246_, C232D_204_ and K234E_206_ in hH may further stabilise the hydrophobic core of the B/C barrel (Fig. 5A). Conserpin harbors two potentially destabilizing mutations, but surrounding mutations have compensatory effects: the known destabilizing mutation F366A_336 _40,55, which in isolation would create a destabilizing cavity, is compensated by the mutation V364F_334_ and the introduction of a coordinated salt-bridge network between D256_228_, E257_229_, K368R_338_ and N367D_337_ (Fig. 5B); the potentially destabilizing mutation W238K_210_, which would likely weaken hydrophobic packing and introduce a large cavity, is offset by backbone polar contacts with E363_333_, and together with I229Y_201_ and A284V_256_ may function as a solvent barrier that shields the hydrophobic core (Fig. 5B). MD simulation also indicates a transient salt bridge between W238K_210_ and D256_228_. Conserpin also contains L224K_196_ and S285E_257_, which staples s2C and s3C together, further stabilizing the native state (Fig. 5B). Finally, L241E_213_ and N228Y_200_ are close to the B-sheet hydrophobic core and the region in which citrate was found to bind and stabilize α1-AT, thus potentially providing extra stability^93^. Taken together, these features may contribute to core nucleation rates during early protein folding, as well as native state resistance to unfolding, consistent with our unfolding and refolding data (Fig. 1).

### Conserpin is less frustrated than α1-AT

**Figure 6:**
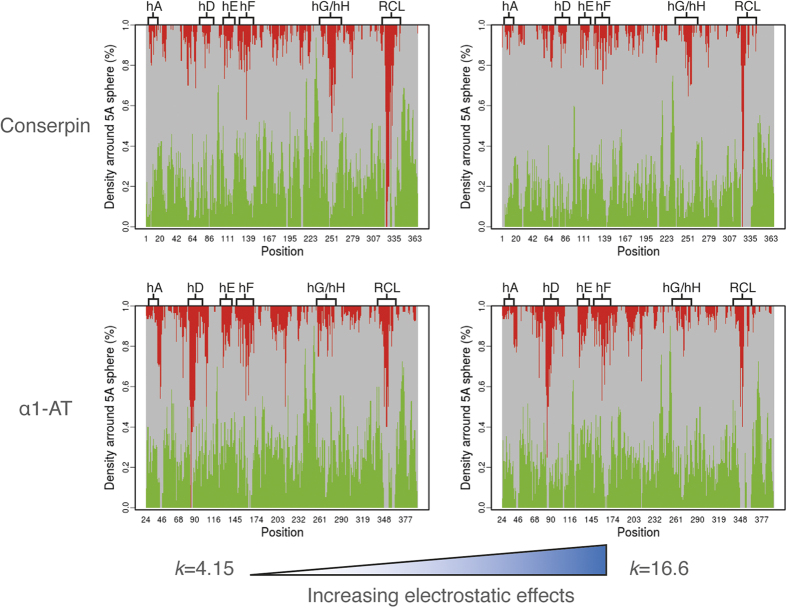
Configurational frustration analysis for conserpin and α1-AT. Minimal, neutral and highly frustrated contacts are represented in green, gray and red respectively. Calculations were performed with different electrostatic strengths by varying the electrostatic constant (*k*). According to ref 95, larger *k* values are related to stronger effects of the Debye–Hückel term.

We next investigated the distribution of energetic frustration within the structures of conserpin and α1-AT using the frustratometer webserver^94^,^95^. As proteins are thought to be minimally frustrated polymers with rugged energy landscapes, the degree of energetic frustration is related to the description of the proteins energy landscape^2^,^27^,^94^; that is, a high level of frustration implies flexibility and a more rugged energy landscape. We therefore used configurational frustration analysis which describes interactions with respect to structural decoys that may be encountered during the folding process^94^,^95^. Overall, conserpin is less frustrated than α1-AT in several regions, with the exception of the RCL, which is shown to be more dynamic during MD simulation (Fig. 6 and S4B,C). In combination with MD simulation, these results show an inverse correlation between the degree of frustration and degree of dynamics, which is mediated by electrostatic effects (Figs 6 and S4B,C). By modulating the electrostatic constant (*k*) from 4.15 to 16.6, it becomes apparent that long-range interactions are essential to the reduced frustration of conserpin, but are not as significant in α1-AT (Fig. 6). This is particularly noticeable for helix D and F, which have fewer highly frustrated contacts in conserpin (Fig. 6). The improved folding properties and increased conserpin stability may therefore be related to the higher number of charged residues that stabilise local contacts and introduce repulsion between patches enriched in like charges, which in turn must be correctly oriented in unfolded forms to avoid aggregation^96^.

## Discussion

The puzzle of how the folding polypeptide chain of serpins achieves a metastable native state has proven challenging to solve. Their unusual and complicated mechanism of protease inhibition challenges the characterization of their folding pathway^42^. It has been established that α1-AT, ACH and PAI-1 all unfold from their native states via an aggregation-prone intermediate ensemble^19^,^22^,^40^,^42^,^43^,^47^,^53^,^55^,^89^,^97^. More recent studies of α1-AT revealed relatively fast folding of the core B/C sheet β-barrel followed by much slower formation of the central A β-sheet^40^. These observations were consistent with models of how off-pathway α1-AT polymers form due to a folding “race” between the core barrel and the central β-sheet^19^,^24^. However, despite two decades of effort, the aggregation-prone nature and poor refolding properties of serpins have prevented a full, atomic level characterization of their folding landscape.

Our study reports for the first time, the successful engineering of a reversibly folding serpin that is highly resistant to polymerization and aggregation, even after the introduction of the polymerogenic, disease-causing Z-mutation. Structural analysis reveals the presence of many context dependant and stabilising interactions in regions that are known to be important for folding. These include stabilizing interactions around the D-helix, a salt bridge network in the A-sheet that may resist aberrant RCL insertion, optimization of A-sheet hydrophobic core packing, stabilising mutations in the F-helix that may raise the energy barrier for RCL insertion, and improved packing in the B/C barrel. Although some single mutations in these same regions have been reported to stabilise α1-AT, we found that many mutations within conserpin act together cooperatively. The relatively large accessible surface area and solvent inaccessible cavity volume compared to all other serpins are unusual for a thermostable protein, suggesting that stabilization is achieved by highly context-specific interactions. Although structure and dynamics suggest stabilisation of the native state in key regions, this represents a conundrum: how can function be maintained, which requires metastability, flexibility and conformational change, in combination with a high degree of stability?

Biophysical and structural analysis paints a complex picture. Although native conserpin features fewer overall number of polar contacts compared to other metastable serpins, new, specific interactions stabilise a rigidified native state that is less frustrated than α1-AT. The inhibitory activity of conserpin confirms that its native state retains metastability required for function. The slightly increased SI of conserpin is most likely due its sub-optimal RCL sequence hindering association with the target protease. However, functional impairment might also be caused by a slowing of the rate of insertion of its RCL into the central A β-sheet, which may also contribute to its aggregation resistance. The latent state structure reveals an overall increase in H-bonds and salt bridges. These observations underline the functional importance of maintaining the relative stabilities of the native versus RCL-inserted state (latent/cleaved) irrespective of the specific stabilising features, as observed previously for thermostable serpins^63^,^64^. However, the structural data do not completely explain the remarkable stability of conserpin. Indeed, the most interesting properties are seen during folding and on exposure to heat; conserpin folds in a concerted fashion, with a relatively minimised population of the aggregation prone intermediate ensemble, and avoids aggregation on heating, with preferential transition to the latent state. Taken together, these results reveal two distinct effects from chemical denaturation and thermal treatment that uniquely provides conserpin with two-state reversible folding, a high degree of thermostability and aggregation resistance. Furthermore, our findings suggest that the robustness of conserpin folding is due to remodelling of its energy landscape, specifically the smoothing of rugged features that trap aggregation-prone intermediates.

Remodelling of the energy landscape is fascinating from an evolutionary and protein engineering perspective. Consensus design typically accumulates residues important to native state stability^28^,^29^,^31^,^32^,^34^,^98^, but could equally alter the folding landscape^37^,^38^,^99^. As such, conserved features of the energy landscape would be solidified or even amplified, whilst non-conserved features would be minimised. For conserpin, this mechanism implies that aggregation and off-pathway folding events are not conserved across the serpin family, possibly because the functional and regulatory requirements of divergently evolved serpin clades sculpted rugged landscapes as an unfortunate consequence, as may be the case for other functional proteins^1^,^2^. Therefore, consensus design can potentially smooth the “risky”, rugged folding landscapes of functional proteins. This may offer several benefits for protein engineering in general, including the removal of aggregation-prone intermediates and modifying protein scaffolds for use as protein therapeutics and diagnostic reagents. In the case of serpins, our structural and folding data for conserpin and Z-conserpin demonstrate the potential of this engineered scaffold as a model system for studying pathological disease mutations. Finally, the fragile nature of serpin folding has thus far hindered residue-level kinetic characterization of all species on the folding pathway, for example using phi-value analysis^100^; the robustness of conserpin may finally provide the basis for such characterization.

## Materials and Methods

To design *conserpin* we used a previously reported MSA of 219 serpin sequences^39^ to generate a consensus sequence as described previously^37^. After filtering to remove incomplete sequences and the application of redundancy reduction, we aligned 212 sequences and generated a new protein sequence by selecting the most frequently observed residue at each column of the MSA (the ‘consensus method’) (Dataset S1). Protein expression and purification, SI measurement and spectroscopic analysis was performed as described previously^89^. Protein Crystallography was performed at the MX1 and MX2 beamlines at the Australian Synchrotron^101^. All experimental and computational methods are described in detail in SI Methods.

## Additional Information

**Accession Numbers:** The coordinates and structure factors have been deposited in the Protein Data Bank under accession codes 5CDX, 5CDZ and 5CE0.

**How to cite this article**: Porebski, B. T. *et al*. Smoothing a rugged protein folding landscape by sequence-based redesign. *Sci. Rep.* **6**, 33958; doi: 10.1038/srep33958 (2016).

## Supplementary Material

Supplementary Information

Supplementary Information
