# Supplementary material for: Smoothing a rugged protein folding landscape by sequence-based redesign
Source: Sci Rep. 2016 Sep 26;6:33958. doi: 10.1038/srep33958 (PMC5036219; doi:10.1038/srep33958)
Supplement: Supplementary Information [file srep33958-s1.pdf]

Supporting Information

Smoothing a rugged protein folding landscape by sequence-based redesign

Benjamin T. Porebski, Shani Keleher, Jeffrey J. Hollins, Adrian A. Nickson, Emilia M. Marijanovic, Natalie A. Borg, Mauricio G.S. Costa, Mary A. Pearce, Weiwen Dai, Liguang Zhu, James Irving, David E. Hoke, Itamar Kass, James C. Whisstock, Stephen P. Bottomley, Geoffrey I. Webb[e], Sheena McGowan, Ashley M. Buckle

SI Figures

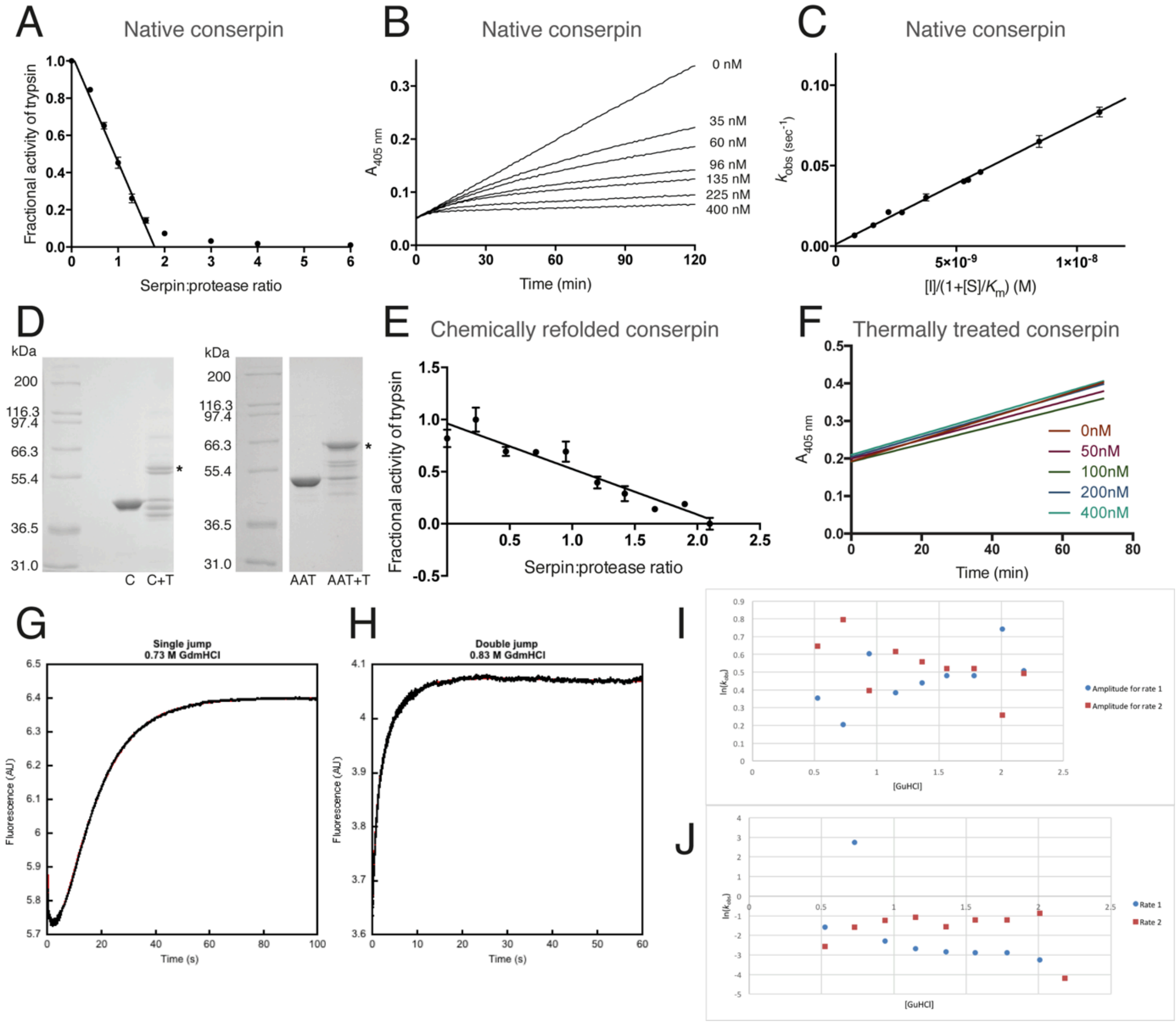

Fig. S1. Activity of conserpin against trypsin. (A) SI (mean and standard error shown, n=6). **(B)** Representative traces of progress curves of trypsin activity (100 nM) in the presence of conserpin. The concentration of conserpin (inhibitor) is indicated next to each curve. $A_{405\ nm}$ is the absorbance measured at 405 nm (arbitrary units). **(C)** Rates of

the progress curves were determined (observed rate constant, k_{obs}). k_{obs} for each inhibitor concentration [I] is plotted against $[I]/(1+[S]/K_m)$ to find k_{ass} (mean and standard error shown, n=3). K_m is the Michaelis constant for trypsin cleavage of the substrate (S). The slope of the linear function was taken as the k_{assapp}, which was then multiplied by the SI to give the k_{ass}. **(D)** Formation of a serpin-protease complex and other species seen in SDS-PAGE formed at a ratio of 2:1 (serpin:protease) under reducing conditions. From left to right. molecular weight markers (kDa); trypsin alone (T); conserpin alone (C); conserpin incubated with trypsin (C+T); α1-AT alone (AAT); α1-AT incubated with trypsin (AAT+T). The serpin-protease complex is labeled as *. Partial degradation of both serpin-protease complexes is observed and likely due to excess trypsin. The conserpin-trypsin complex has an expected MW of 62.4 kDa consisting of cleaved conserpin (38.8 kDa) and trypsin (23.8 kDa). Conserpin-trypsin also displayed higher molecular weight species not seen in the inhibition of trypsin by α1-AT. **(E)** SI (mean and standard error shown, n=3) of conserpin after chemical denaturation in 6 M GuHCl and rapid dilution into TBS. **(F)** Representative traces of progress curves of trypsin activity (100 nM) in the presence of conserpin that has been heated at 80°C for 20 minutes. Conserpin (inhibitor) concentrations are indicated. $A_{405\ nm}$ is the absorbance measured at 405 nm (arbitrary units). **(G)** Single jump kinetics trace of Conserpin upon mixing with 0.73 M GuHCl. **(H)** Double jump kinetics trace of Conserpin upon mixing with 0.83 M GuHCl. **(I)** Single jump relative amplitudes of conserpin reveal two distinct refolding rates (blue circles and red squares). **(J)** Single jump rate constants of conserpin reveal two distinct refolding rates.

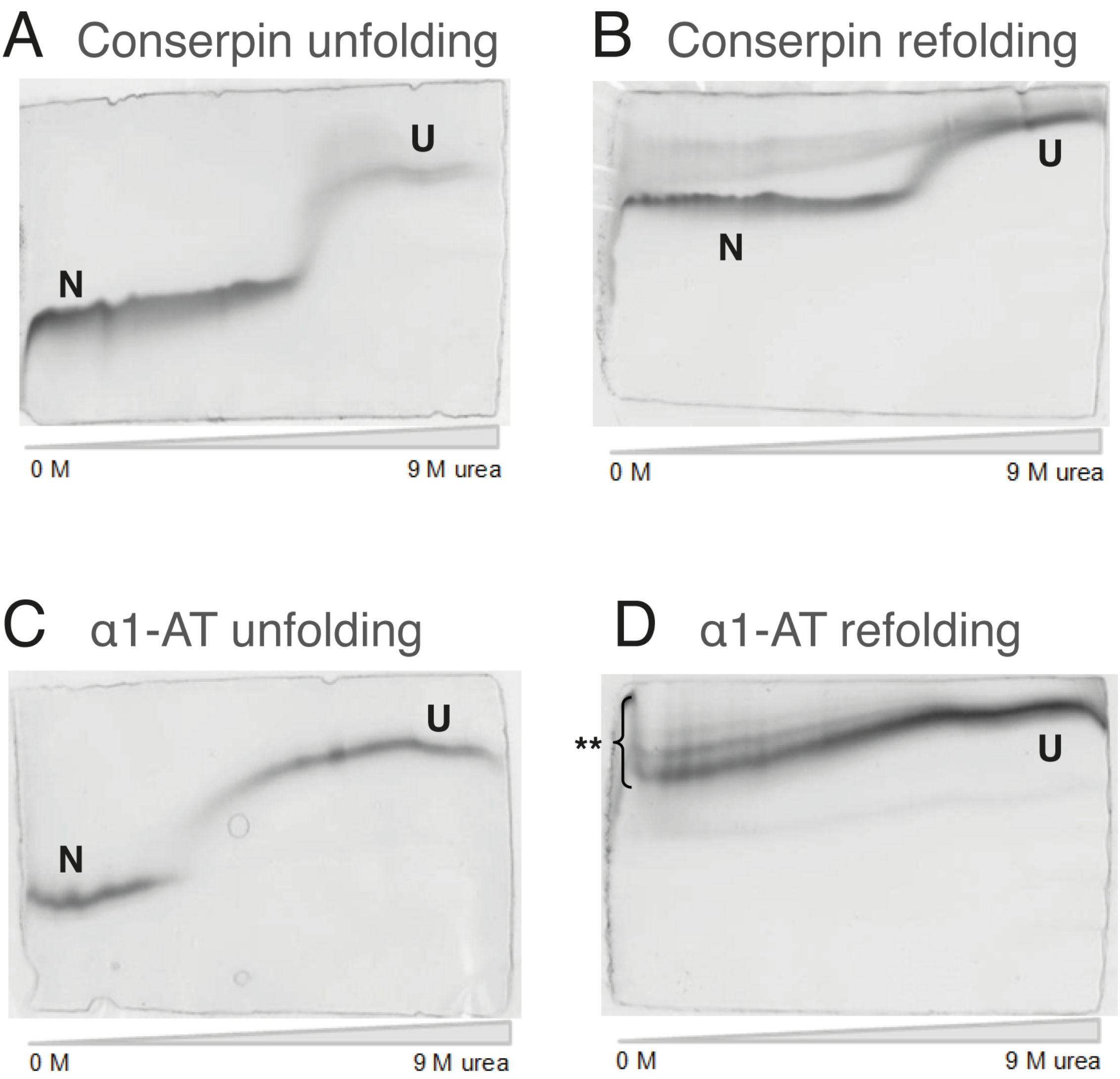

Fig. S2. TUG gels show that conserpin is more stable than α1-AT in urea and more resistant to polymerization upon refolding. The **(A)** unfolding and **(B)** refolding of conserpin, and the **(C)** unfolding and **(D)** refolding of α1-AT. The TUG gels contain a gradient of 0 M to 9 M urea from left to right. N indicates the native species, U indicates the unfolded species and ** indicates polymers. α1-AT polymers can be seen as laddering of higher molecular weight species near the top of the gel. Differences between the position of N in **(A)** and **(B)** are the result of non-equal run time of the gel.

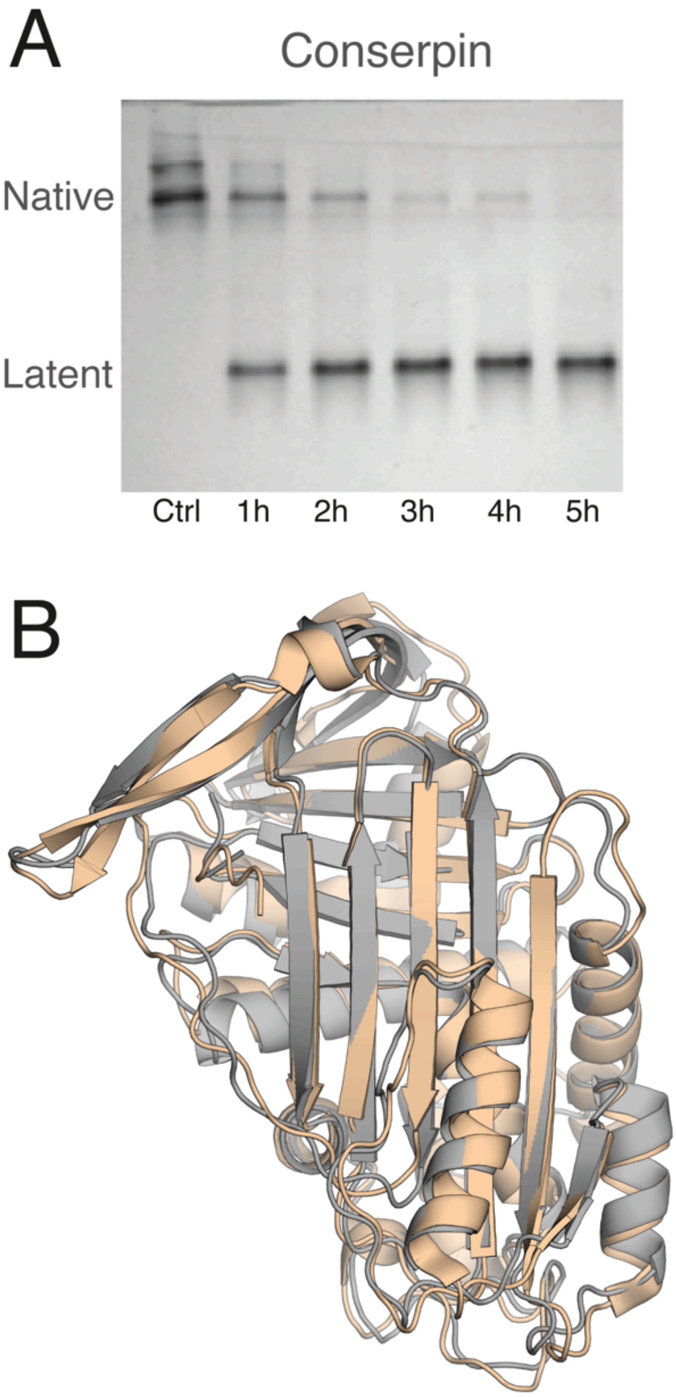

Fig. S3. Formation and structure of latent conserpin. (A) Native gel containing 9 M urea after conserpin is heated at 76 °C for 1 to 5 h. **(B)** Structural alignment of latent conserpin (grey) and latent α1-AT (wheat; PDB: 1IZ2). The structure reveals successful adoption of the latent conformation, with the RCL inserted between s3A and s5A.

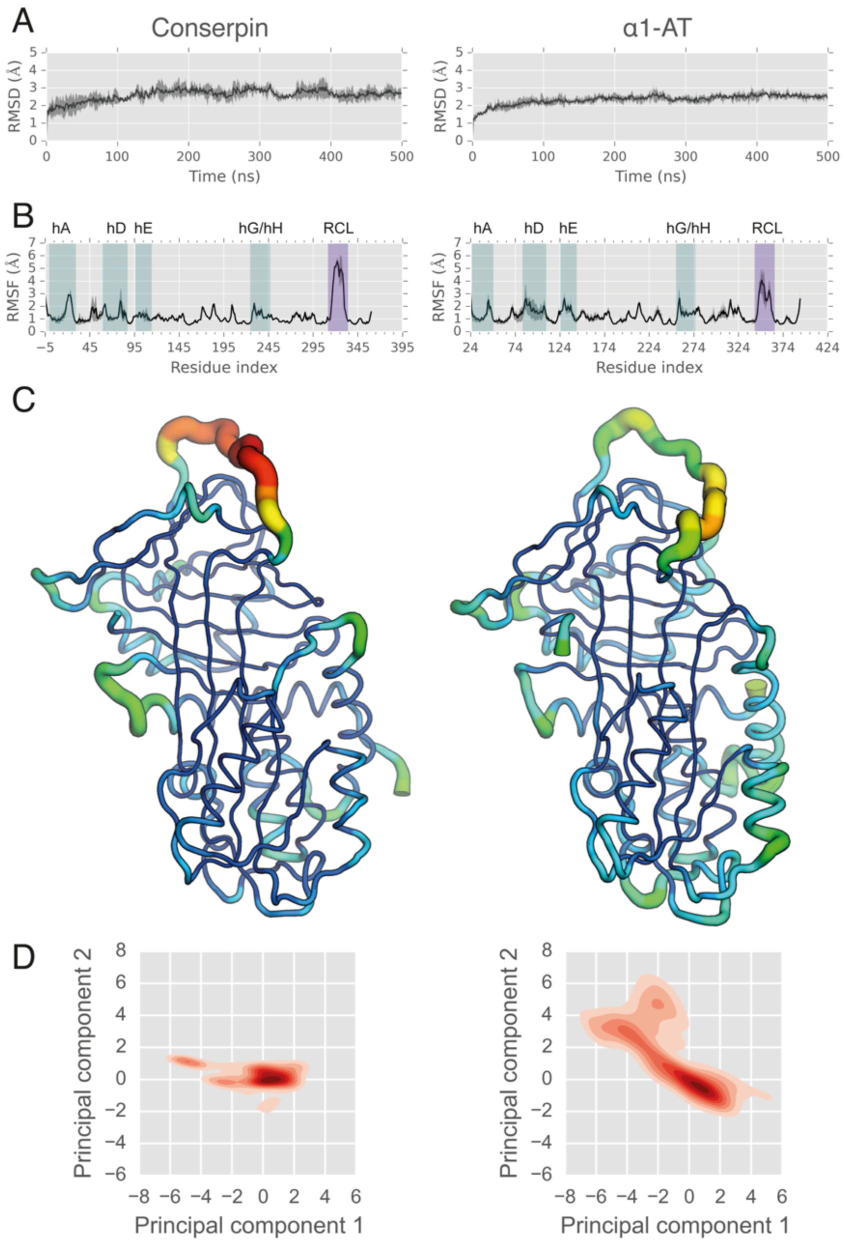

Fig. S4. Molecular dynamics simulation of conserpin and α1-AT. (A) Root mean square deviation (RMSD) plots of Cα atoms in conserpin (average RMSD of 2.60 Å) and α1-AT (average RMSD of 2.34 Å) over 500 ns at 300 K. Plots show the mean RMSD (solid line) with the min/max variation (n=2). **(B)** Root mean square fluctuation (RMSF) plots of Cα atoms in conserpin and α1-AT over 500 ns at 300 K (n=2). **(C)** Conserpin (left) and α1-AT RMSF values of each Cα atom embedded onto their corresponding crystal structures as B-factor putty. The width of the tubes and their colors (blue to red) are proportional to the magnitude of the RMSF (maximum: 6 Å). **(D)** Principal component analysis (modes 1 and 2) of all MD simulation replicates reveals the substantially reduced conformational sampling by conserpin.

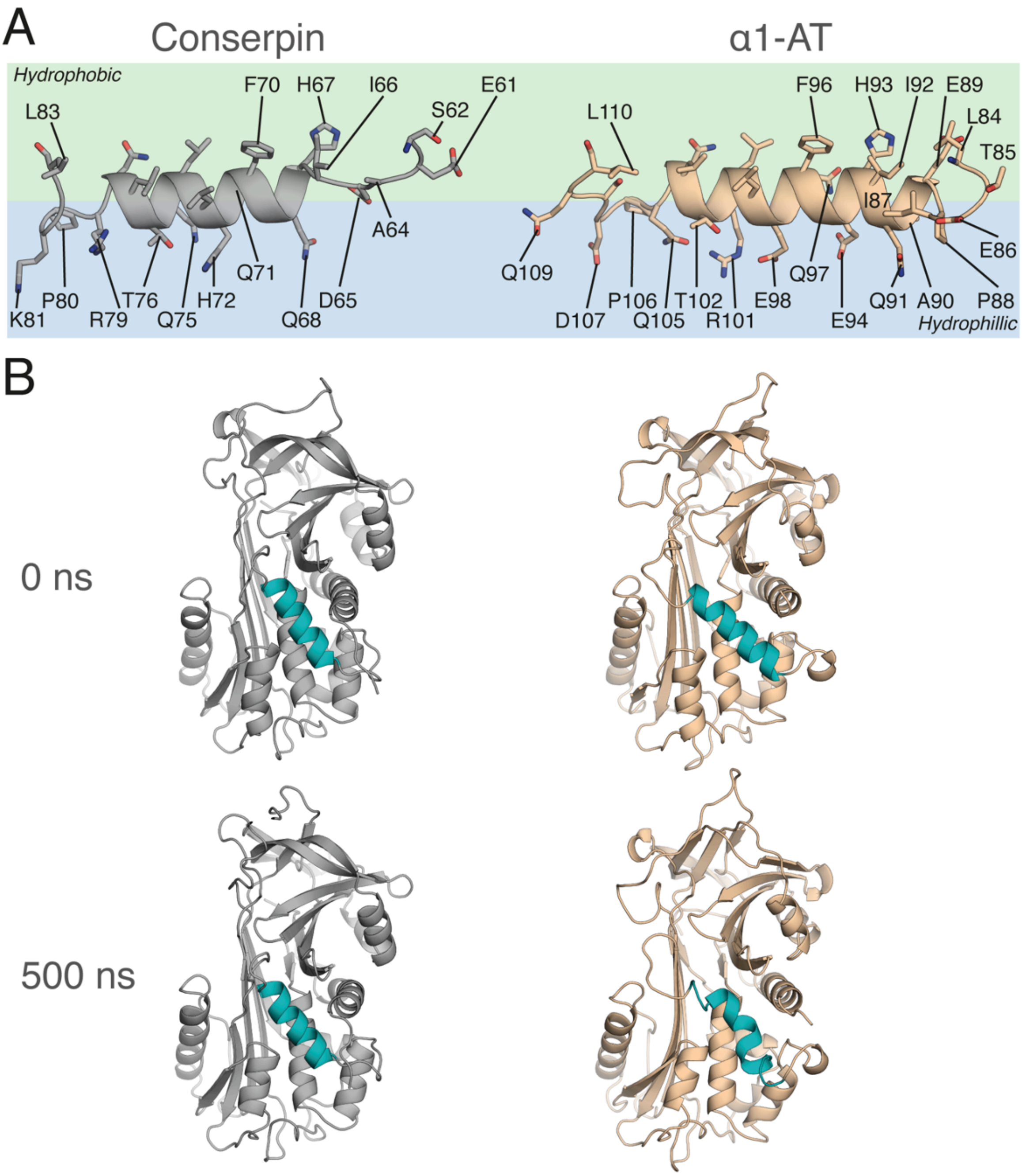

Fig. S5. Inspection of the D-helix. (A) Differences in the D-helix between conserpin (grey) and α1-AT (wheat). The hydrophobic (green) section faces and packs against the hydrophobic core, whilst the hydrophilic (blue) section faces into solvent. **(B)** Partial loss of structure in the D-helix (cyan) of α1-AT after 500 ns of MD.

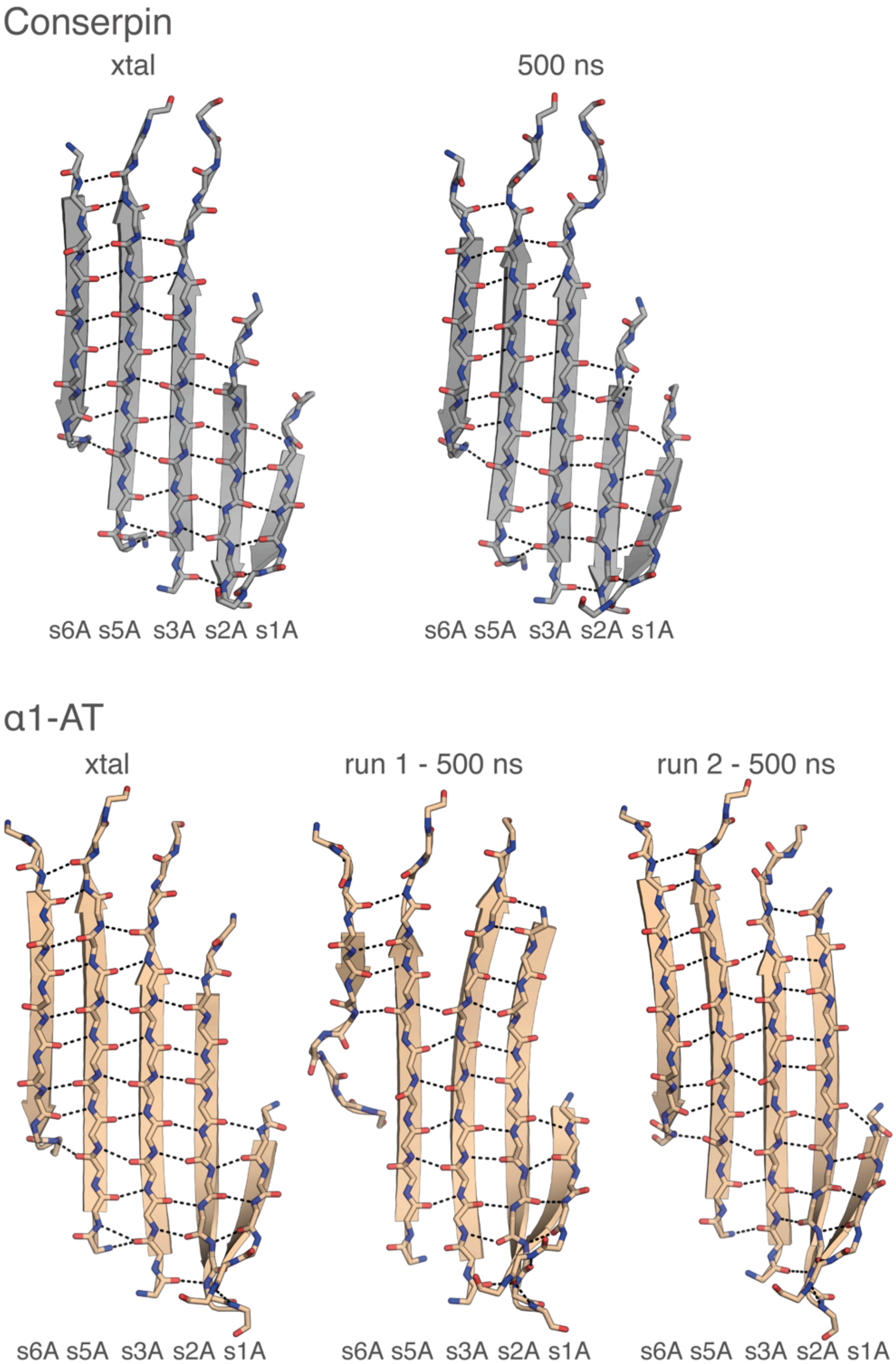

Fig. S6. Hydrogen bonding differences between the backbone of sheet-A in conserpin (grey) and α1-AT (wheat) from crystal structure and 500 ns of MD, highlighting the increased propensity for separation between s3A and s5A in α1-AT.

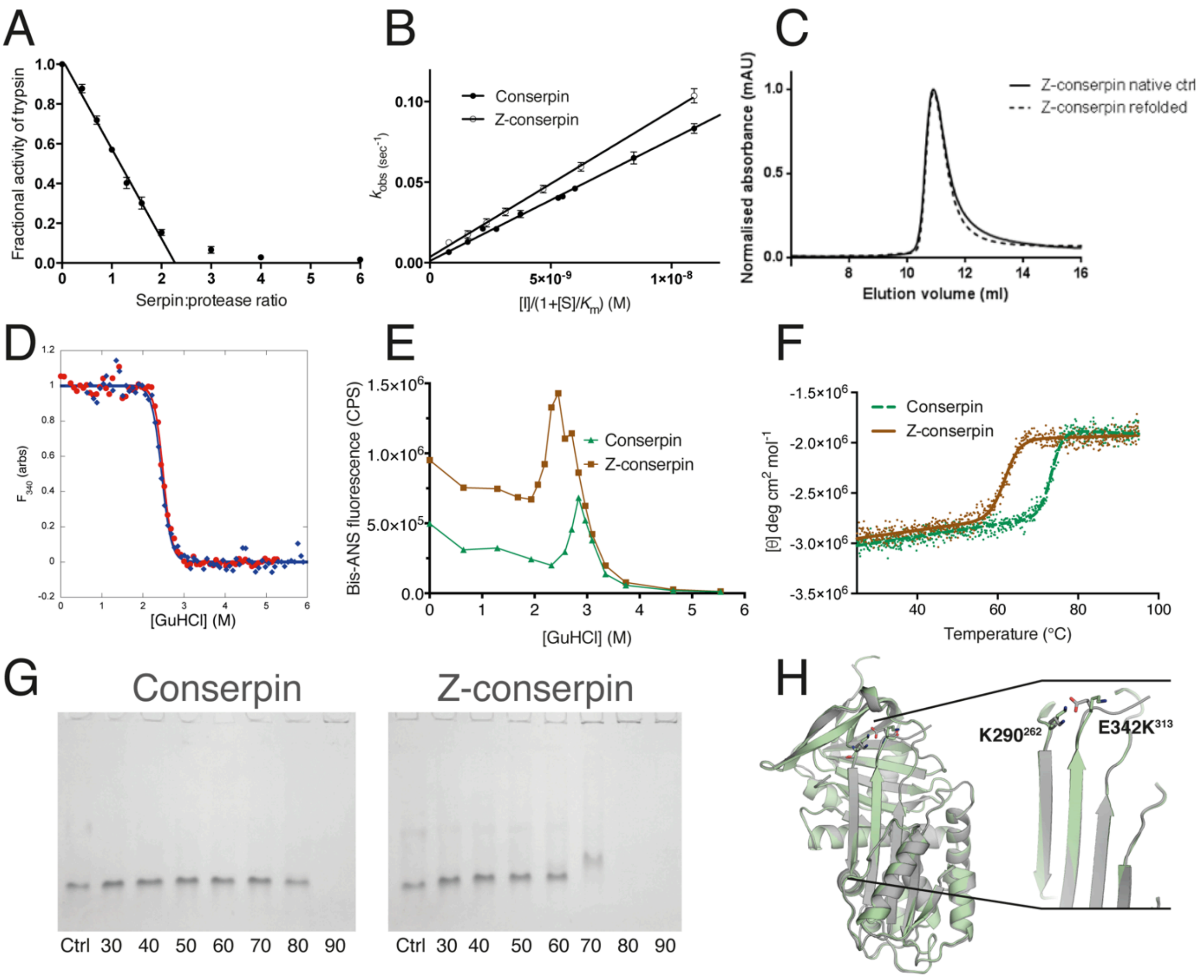

Fig. S7. Function, folding, polymerization and structure of Z-conserpin. (A) SI (mean and standard error shown, n=6) of Z-conserpin against trypsin. **(B)** k_{obs} for each inhibitor concentration [I] is plotted against $[I]/(1+[S]/K_m)$ to find k_{ass} (mean and standard error shown, n=3). K_m is the Michaelis constant for trypsin cleavage of the substrate. The slope of the linear function was taken as the k_{assapp}, which was then multiplied by the SI to give the k_{ass}. **(C)** Gel filtration of native and refolded Z-conserpin as described in Fig. 1. **(D)** Equilibrium unfolding and refolding of Z-conserpin using intrinsic fluorescence at 280 nm. **(E)** Equilibrium unfolding of Z-conserpin under the presence of bis-ANS. **(F)** Thermal unfolding of conserpin and Z-conserpin in 2M GuHCl. **(G)** Native PAGE of conserpin (left) and Z-conserpin (right), where samples were heated for 10 mins from 30C to 90C. **(H)** Structural alignment of conserpin (grey) and Z-conserpin (pale green), with a close up alignment, highlighting rotamer differences at E342K^{313}, resulting in the loss of a salt bridge.

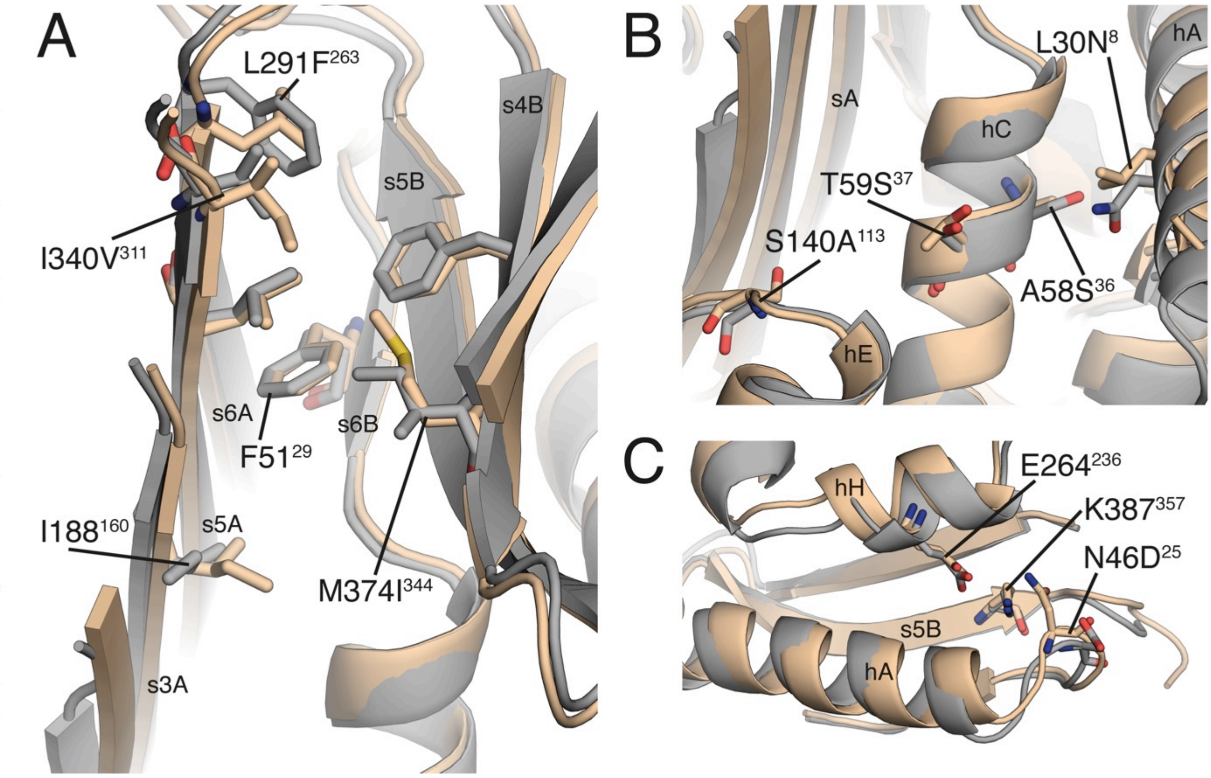

Fig. S8. A-sheet hydrophobic core interactions in conserpin (grey) and α1-AT (wheat). (A) Comparison of the hydrophobic core surrounding F51[29] in conserpin and α1-AT. Adjacent mutations I340V[311] and L291F[263], M374I[244], and an alternate conformation of I188[160] optimize packing of the hydrophobic core in conserpin. **(B)** Comparison surrounding the T59S[37] mutation in conserpin, which is surrounded by additional mutations L30N[8], A58S[36] and S140A[113], together allowing for favorable non-polar and polar interactions that are not possible in α1-AT. Similarly, L291F[263] improves van der Waals packing against s6A (Panel A). **(C)** Comparison surrounding K387[357], showing the salt bridge introduced by the N46D[25] mutation. Both α1-AT and conserpin contain K387[357], buried in the core. However, in conserpin the neighboring mutation N46D[25] allows for the formation of a transient salt bridge between E264[236] of hH and K387[357] of s5B during MD.

Table S1: *Data collection and refinement statistics.*

Data collection	Native conserpin	Latent conserpin	Native Z-conserpin
Wavelength (Å)	0.9537	0.9537	0.9537
Space group	C 2 2 2_1	P 62	P 1 2_1 1
Unit cell dimensions (Å)	68.14, 76.12, 150.24, 90, 90, 90	101.679, 101.679, 62.72, 90, 90, 120	49.22, 150.27, 51.015, 90, 94.74, 90
Resolution (Å)	2.4	1.449	2.3
Measured reflections	30073	2493126	252442
Unique reflections	15325	65513	32616
Completeness (%)	97.77	99.94	99.64
Redundancy	2.0	38.0	7.7
R_{pim}	0.04451		
<I/σI>	9.20	151.41	30.83
Structure refinement			
Number of reflections	15304	65500	32565
Number of protein atoms	2647	2853	5345
Number of water molecules	58	351	243
R_{work} (%)	0.1942	0.1566	0.2012
R_{free} (5% of data) (%)	0.2582	0.1851	0.2294
CC1/2	0.997	0.826	0.99
CC*	0.999	0.951	0.997
RMSD bond lengths (Å)	0.007	0.012	0.003
RMSD bond angles (º)	1.03	1.57	0.69
Average B-factor (Å^2)	55.10	25.30	51.00
Protein	55.20	23.60	51.20
Solvent	52.30	38.80	46.40
Ramachandran			
Favoured (%)	97	97	98
Outliers (%)	0	1.4	0
MolProbity score	1.65, 99th percentile (N=8058, 2.40Å ± 0.25Å)	1.56, 78th percentile (N=3441, 1.449Å ± 0.25Å)	1.18, 100th percentile (N=8909, 2.30Å ± 0.25Å)
PDB ID	5CDX	5CDZ	5CE0

Table S2: Global physiochemical properties of serpins in available native, latent and cleaved state structures.

**Serpin**	**PDB code**	***Tm***	**H-bonds**	**Salt bridges < 7 Å**	**Accessible surface area (Å²)**	**Total cavity volume (Å³)**	**Reduction (ASA%, TCV%)**
$\text{Conserpin}_{\text{Native}}$	5CDX	>110°C*	178	112	17,280.7	2,257.6	
$\text{Conserpin}_{\text{Latent}}$	5CDZ	N/A	200	102	16,101.6	1,216.2	6.9%, 46.1%
$\alpha\text{1-AT}_{\text{Native}}$	3NE4	61.8°C	206	126	16,599.3	1,928.6	
$\alpha\text{1-AT}_{\text{Latent}}$	1IZ2	N/A	194	112	16,284.3	873.6	1.9%, 54.7%
$\alpha\text{1-AT}_{\text{Cleaved}}$	1D5S	N/A	190	117	17,103.1	1,190.8	-3.0%, 38.3%
$\text{Thermopin}_{\text{Native}}$	1SNG	67°C	178	207	16,852.1	1,279.8	
$\text{Thermopin}_{\text{Cleaved}}$	1MTP	N/A	226	225	14,009.3	547.7	16.9%, 57.2%
$\text{Tengpin}_{\text{Native}}$	2PEE	N/A	207	91	16,667.1	1,844.1	
$\text{Tengpin}_{\text{Latent}}$	2PEF	N/A	212	90	16,345.7	1,112.1	1.9%, 39.7%
$\text{PAI-1}_{\text{Native}}$	1OC0	43-52°C	209	117	16,323.6	1,076.9	
$\text{PAI-1}_{\text{Latent}}$	1DVN	68°C	214	144	15,639.2	602.6	4.2%, 44.0%
$\text{ACH}_{\text{Native}}$	1YXA		192	121	17,573.9	1,220.3	
$\text{ACH}_{\text{Cleaved}}$	2ACH		196	111	15,314.2	656.8	12.9%, 46.2%
$\text{Neuroserpin}_{\text{Native}}$	3FGQ	45°C	217	151	16,943.0	969.5	
$\text{Neuroserpin}_{\text{Cleaved}}$	3F02	N/A	213	143	15,853.5	594.0	6.4%, 38.7%
$\text{Antithrombin}_{\text{Native}}$	2ANT		206	188	18,570.7	1592.9	
$\text{Antithrombin}_{\text{Latent}}$	2ANT		205	153	17,695.0	1033.1	4.7%, 35.1%

* A true melting temperature (*Tm*) was not determined in our experiments. All other values were determined as per SI methods.

SI Methods

Design of conserpin. Conserpin was designed from an alignment of 212 sequences within the serpin superfamily; this alignment was based on the alignment of 219 serpin sequences produced by Irving *et al.* [1]. In order to remove bias from the alignment, we removed redundant sequences above 95% similarity using CD-HIT [2], which resulted in 212 sequences. Application of a 100% consensus algorithm was applied over all 212 sequences (Dataset S1), resulting in a single sequence, which we call *conserpin*.

Protein expression and purification. The conserpin gene was synthesized by DNA 2.0 (CA, USA) and cloned into a pLIC-His vector using Ligation Independent Cloning. Proteins were expressed in *Escherichia coli* BL21 (DE3) using autoinduction media *(Overnight Express Instant TB* Medium, Novagen) at 28 °C until an OD_{600} of ≥10 was obtained. Cells were harvested by centrifugation and resuspended in lysis buffer (25 mM NaH_2PO_4, 300 mM NaCl, pH 8.0; 2 mM β-mercaptoethanol, PMSF and lysozyme). Cells were disrupted using sonication and whole cell lysate collected via centrifugation. 10 mM imidazole was added to the collected whole cell supernatant and applied to a 5 ml His Trap HP nickel-affinity column (GE Healthcare, UK). The column was washed with 20 mM imidazole in lysis buffer before bulk elution with 250 mM imidazole. Conserpin was further purified using size-exclusion chromatography (Superdex 200 16/60, GE Healthcare, UK) in 50 mM Tris, 90 mM NaCl, pH 8.0. Purified conserpin was concentrated to ~ 200 μM, as measured using a NanoDrop ND-1000 spectrophotometer (Thermo Scientific, DE, USA), and stored at -80 °C. Expression of conserpin was verified using mass spectrometry (MS) with a Matrix-Assisted Laser Desorption Ionization Time-of-Flight Time-of-Flight (MALDI-TOF-TOF). Approximately 10 mg of pure monomeric protein was obtained from a 250 ml autoinduction culture. Z-conserpin was created by QuikChange method (Stratagene) against the conserpin plasmid using KOD DNA polymerase (Novagen). Oligonucleotides were synthesized by Geneworks (Australia). Z-conserpin was expressed and purified using the procedure outlined above. Wild-type α1-AT was purified from *E. coli* as per [3]. Z-α1-AT was purified from *P. pastoris* as previously described [4].

Gel filtration analysis of protein refolding. Samples of native and refolded protein were analyzed using a Superdex 75 10/300 column (GE Healthcare, UK) in 50 mM Tris, 90 mM NaCl, pH 8.0 and a 280 nm lamp. For refolding, the protein was unfolded for 1 h in 5 M GuHCl and then refolded by performing a 10 times dilution of the sample. Final samples were at a concentration of 2 μM and were centrifuged for 5 min to remove large protein aggregates before loading onto the column using a 500 μl loop.

Native PAGE. 5 μl samples of protein at a concentration of 10 μM in 50 mM Tris, 90 mM NaCl, pH 8.0 were heated at various temperatures in a heating block and immediately placed on ice and mixed with ice-cold non-denaturing loading buffer to be run on 10 %

(w/v) acrylamide native gels with a discontinuous buffer system. 9 M urea native gels were prepared as 10 % (w/v) native gels but with urea dissolved into the running gel to a final concentration of 9 M.

Transverse Urea Gradient (TUG) gel analysis. TUG gel analysis was performed as previously described [5], using a 0–9 M urea gradient perpendicular to the direction of electrophoresis. The running buffer used was 43 mM imidazole, 35 mM HEPES (pH 7.2-7.8). The unfolding of the protein was examined by applying native protein to the gel, and refolding by applying protein pre-denatured in urea.

Determination of protease inhibition kinetic parameters. The stoichiometry of inhibition (SI) and rate of association (k_{ass}) were measured at 37 °C in 50 mM Tris, 150 mM NaCl, 0.2% (w/v) PEG 8000, pH 7.4 as previously described [6]. Conserpin was incubated with trypsin for 30 min before measuring residual trypsin activity to calculate the SI. Trypsin (T1426, Sigma-Aldrich, MO, USA) activity was measured using the substrate S-2222 (Chromogenix, Italy). When measuring progress curves to calculate k_{ass} the final concentration of trypsin was 0.04 nM. For complex gels conserpin was incubated with trypsin in a 2:1 molar ratio of serpin:protease for 30 min at 37 °C in 50 mM Tris, 150 mM NaCl, 0.2% (w/v) PEG 8000, pH 7.4.

Characterization of Thermal Stability. Thermal stability of purified serpins was measured by circular dichroism. CD measurements were performed using a Jasco 815 spectropolarimeter; 0.2 mg/ml protein in PBS (140 mM NaCl, 2.7 mM KCl, 10 mM PO_4^{3-}, pH 7.4) was used in a 0.1 cm path length cuvette. Thermal denaturation was measured by observing signal changes at 222 nm during heating at a rate of 1 °C/min. The T_m was obtained by fitting to a sigmoidal dose-response (variable slope) equation. Raw CD data was converted from milidegrees to molar ellipticity [θ] via the following equation:

$$[\theta] = \frac{\theta \times MW}{10 \times C \times L}$$

Where θ is the raw data in milidegrees, MW is the average protein molecular weight in g/mol, C is the protein concentration in g/L and L is the cuvette path length of 0.1 cm.

Equilibrium Measurements. A 6 M solution of guanidine hydrochloride (GuHCl) in TBS was combined in varying ratios with TBS buffer using a liquid handling robot to create a range of denaturant solutions from 0 – 6 M GuHCl. These solutions were subsequently mixed in an 8:1 ratio with 9 μM protein in TBS to give a final concentration of 1 μM protein. All solutions were left to equilibrate at 25°C for at least three hours, after which the fluorescence of each solution was measured on a Perkin Elmer LS55 fluorimeter using an excitation wavelength of 280 nm and an emission range of 300 – 400 nm. Readings were obtained from a 1 cm pathlength cuvette maintained at 25 ± 0.1°C. The experiment was repeated, but using 9 μM protein pre-unfolded in 6 M GuHCl to generate a refolding curve. These solutions were left to equilibrate for at least six hours before their fluorescence was ascertained. Bis-ANS unfolding experiments were conducted in a similar manner, except with the addition of bis-ANS to a final concentration of 5 μM.

Kinetic Measurements. Folding was monitored by changes in fluorescence using a 350 nm cut-off filter and an excitation wavelength of 280 nm. All experiments were performed using an Applied Photophysics (Leatherhead, UK) stopped-flow apparatus maintained at 25 ± 0.1°C. For unfolding experiments, one volume of 11 μM protein solution was mixed rapidly with ten volumes of a concentrated GuHCl solution. For single jump refolding, the protein (11 μM) was unfolded in 6 M GuHCl and left to equilibrate for at least 30 mins before use. This was then mixed 10:1 with buffer to give a final concentration of 1 μM, resulting in some very complex traces. To correct this, we conducted double jump refolding, where the protein (12 μM in 2M GuHCl) was diluted 1:1 with 6 M GuHCl buffer (to give 6 μM in 4 M GuHCl). This solution was left for a variable delay time (1 —> 50 s) and was then mixed 1:5 with buffer (to give 1 μM in 0.67 M GuHCl). Double jump resulted in much cleaner traces and allowed for data fitting to a double exponential. Data collected from at least six experiments were averaged and traces were fit to a single (unfolding) double (double jump refolding) or triple (single jump refolding) exponential as appropriate. Due to mixing effects, data collected in the first 2.5 ms were always removed before fitting.

Data analysis of equilibrium and kinetic measurements. An Excel spreadsheet was used to derive the fluorescence average emission wavelength (AEW) for each of the equilibrated denaturant solutions. A plot of AEW against denaturant concentration

(Kaleidagraph, Synergy Software) yielded the expected sigmoidal plot, which was fitted to a standard two-state equation to obtain the *m*-value ($m_{D\text{-}N}$), the denaturant activity 50% ($[D']_{50}$) and hence the stability of the protein in TBS buffer ($\Delta G_{D\text{-}N}$). Both the unfolding and refolding AEW curves can be converted to Fraction Folded by first removing the baselines and then normalizing the resulting data.

All kinetic traces fitted well to a single exponential decay plus a linear drift term. Single jump refolding yielded at least three different phases, which made data analysis problematic. We suspect that at least one of these (the slowest) phases may be a proline phase since it disappeared in the double jump experiments. The resulting chevron plot from our double jump experiments showed rollover in the refolding arm (indicating the presence of a refolding intermediate) and a kink in the unfolding arm (indicating the presence of a high energy intermediate). Double jump experiments also identified the presence of a second refolding rate that was distinguished by amplitude analysis. The main rate was then fitted using Prism (Synergy Software) to the following equation to estimate all parameters:

$$\ln(k_{\mathrm{obs}}) = \ln\left(\frac{1}{2}\left(-A_1 - \sqrt{{A_1}^2 - 4A_2}\right)\right)$$

where:

$$A_1 = -\left(k_f + k_{-1}e^{m_{-1}[D]} + k_2 e^{-m_2[D]} + k_{-2}e^{m_{-2}[D]}\right)$$
$$A_2 = \left(k_f\left(k_2 e^{-m_2[D]} + k_{-2}e^{m_{-2}[D]}\right) + k_{-1}e^{m_{-1}[D]} + k_{-2}e^{m_{-2}[D]}\right)$$
$$k_f = k_i e^{-m_i[D]}\left(\frac{1}{1 + \frac{k_i e^{-m_i[D]}}{k_d e^{-m_d[D]}}}\right)$$

k_i and m_i are the folding rate constant from the refolding intermediate (I) to the first transition state (TS1) and its associated *m*-value, k_d and m_d are from the denatured state (D) to the first transition state (TS1), k_{-1} and m_{-1} are unfolding from the high energy intermediate (I*) over TS1, k_2 and m_2 are folding from the high energy intermediate (I*) over TS2, k_{-2} and m_{-2} are unfolding from the native state (N) over TS2. By convention, k_{-1} is set as 100,000 s^{-1} and m_{-1} is set as 0 M^{-1}: *m*2 is thus the *m*-value between TS1 and TS2 while the ratio k_{-1}/k_2 informs on the difference in free energy between the two transition states.

Crystallization, X-ray data collection, structure determination and refinement. All crystals were grown using the hanging drop vapor diffusion method, with 1:1 (v/v) ratio of protein to mother liquor (0.5 ml well volume). For native conserpin, the protein was concentrated to 10 mg/ml. Crystals appeared within 5 days in 0.2 M magnesium chloride hexahydrate, 16% PEG-3350, 10 mM bis Tris, pH 7.5. For native Z-conserpin, the protein was concentrated to 9.6 mg/ml. Large, rectangular crystals appeared within 5 days in 20% (v/v) polyethylene glycol (PEG) 3350, 10 mM bis Tris (pH 7.5) and 0.2 M Magnesium chloride (hexahydrate) and did not grow further. For latent conserpin, the protein was concentrated to 10 mg/ml. Crystals appeared with 5 days in 0.1 M Hepes pH 8.0, 0.2 M Ammonium Acetate, 35% v/v MPD. All crystals were cryo-protected by the addition of 10 % glycerol prior to data collection.

Data for all three crystals was collected at 100 K using at the Australian synchrotron macro crystallography MX1 beamline. A 1.7 Å dataset was collected for conserpin and resolution cut to 2.4 Å, 2.3 Å for Z-conserpin and 1.45 Å for latent conserpin. Diffraction images were processed using iMosflm [7] and Aimless from the CCP4 suite [8]. Each dataset was processed in *P*1 and Laue group determination was achieved using Pointless within Aimless. Datasets were scaled and merged in their respective space-group and 5% of each dataset was flagged for calculation of R_{Free}, with neither a sigma nor a low-resolution cut-off applied to any dataset. A summary of statistics is provided in Table S1.

Structure determination proceeded using the Molecular Replacement method and the program PHASER [9]. A search model for conserpin was constructed from the crystal structure of native α1-AT (PDB: 3NE4) by removing solvent molecules. All other structures used native conserpin as the search model. A single clear peak for both the rotation and translation functions was evident and the molecules packed well within the asymmetric unit. Together with the unbiased features in the initial electron density maps, the correctness of the molecular replacement solutions was confirmed. Automated model building was performed using AutoBuild in the Phenix package [10]. All subsequent model building and structural validation was done using Phenix [10] and COOT [11]. Solvent molecules were added only if they had acceptable hydrogen-bonding geometry contacts of 2.5 to 3.5 Å with protein atoms or with existing solvent and were in good $2F_o$-F_c and F_o-F_c electron density. The coordinates and structure factors are available from the Protein Data Bank (5CDX, 5CE0, 5CDZ).

Structure analysis. For all analysis and MD simulations, missing atoms, side chains and residues were rebuilt using Modeller V. 9.12 [12]. In each instance, 50 models were built and the lowest DOPE (Discrete Optimized Protein Energy) scoring model was selected for further analysis. Hydrogen bonding and salt bridge values were calculated using the WHAT-IF web-server (Hekkelman *et al.,* 2010). Solvent accessible surface area was calculated using AREAIMOL as part of the ccp4 package with a default probe radius of 1.4 Å [8]. Total cavity volumes and related structures were calculated using the Depth web server [13]. Molecular graphics were prepared with PyMol Molecular Graphics, Ver. 1.5.0.4

MD system setup and simulation protocol. Molecular dynamics simulations were carried out on native conserpin and native α1-AT. Missing atoms, side chains and residues were modeled as described above. Chain termini were capped with neutral groups (acetyl and methylamide). Residues were protonated according to their states at pH 7.

Completed structures were solvated in a rectangular simulation box leaving at least 10 Å of water shell thickness on all sides of the protein. System charges were neutralized with respective sodium or chloride counter ions. Protein and ions were modeled using the AMBER ff99SB force field [14] and waters were represented using the 3-particle TIP3P model [15]. All bonds involving hydrogen atoms were constrained to their equilibrium lengths with the SHAKE algorithm [16]. The resulting systems were subjected to at least 10,000 energy minimization steps to remove any clashes, followed by an equilibration protocol. During equilibration, we applied harmonic positional restraints of 10 $kcal^{-1}$ mol^{-1} $Å^{2\ -1}$ to the protein backbone atoms, pressure was kept at 1 atm using Berendsen algorithm [17] and the temperature was increased from 10 K to 300 K as a linear function of time over the course of 1.2 ns, with Langevin temperature coupling. Relaxation times for temperature and pressure were 0.5 ps. Subsequently, we removed the restraints and performed a 5-ns simulation at constant isotropic pressure of 1 atm and temperature of 300 K. Electrostatic interactions were computed using an 8-Å cutoff radius and the Particle Mesh Ewald method for long-range interactions [18]. All MD simulations (equilibration and production) were carried out under periodic boundary conditions.

Production simulations of native α1-AT and conserpin were carried out in the NPT ensemble. Temperature was kept at 300 K using the Langevin thermostat with a

collision frequency of 2 ps, whilst Berensden pressure coupling was used to maintain the pressure at 1 atm with a 2ps relaxation time. The simulation time step was 2 fs and snapshots were taken every 100 ps. Simulations were run in duplicate with Amber 14 [19], using PMEMD on a Nvidia K20m GPU for 500 ns.

MD analysis. Simulation trajectories were processed and analysed using a combination of Amber Tools 14, custom scripts and ProDy [19,20]. Graphs and plots were produced with Matplotlib [21]. Molecular graphics were prepared with PyMol ver. 1.5.3 [22].

Local Frustration Analysis Local frustration analysis was conducted with the Frustratometer web server [23], using a completed model based on the crystal structures of conserpin and α1-AT (PDB: 3NE4) in the native state. Essentially, the energetic frustration is obtained by the comparison of the native state interactions to a set of generated "decoy" states where the identities of each residue are mutated. A contact is defined as "minimally frustrated" or "highly frustrated" upon comparison of its frustration energy with values obtained from the decoy states, as described [24].

References

1. Irving, J. A., Pike, R. N., Lesk, A. M. & Whisstock, J. C. Phylogeny of the serpin superfamily: implications of patterns of amino acid conservation for structure and function. *Genome Research* **10,** 1845–1864 (2000).
2. Huang, Y., Niu, B., Gao, Y., Fu, L. & Li, W. CD-HIT Suite: a web server for clustering and comparing biological sequences. *Bioinformatics* **26,** 680–682 (2010).
3. Chang, W. S. *et al.* Importance of the release of strand 1C to the polymerization mechanism of inhibitory serpins. *Protein Sci.* **6,** 89–98 (1997).
4. Levina, V. *et al.* Expression, purification and characterization of recombinant Z alpha(1)-antitrypsin--the most common cause of alpha(1)-antitrypsin deficiency. *Protein Expr. Purif.* **68,** 226–232 (2009).
5. Dafforn, T. R., Pike, R. N. & Bottomley, S. P. Physical characterization of serpin conformations. *Methods* **32,** 150–158 (2004).
6. Le Bonniec, B. F., Guinto, E. R. & Stone, S. R. Identification of Residues in Thrombin-Modulating Interactions with Antithrombin III and. alpha. 1-Antitrypsin. *Biochemistry* **34,** 12241–12248 (1995).
7. Battye, T. G. G., Kontogiannis, L., Johnson, O., Powell, H. R. & Leslie, A. G. W. iMOSFLM: a new graphical interface for diffraction-image processing with MOSFLM. *Acta Crystallogr. D Biol. Crystallogr.* **67,** 271–281 (2011).
8. Winn, M. D. *et al.* Overview of the CCP4 suite and current developments. *Acta Crystallogr. D Biol. Crystallogr.* **67,** 235–242 (2011).
9. McCoy, A. J. *et al.* Phaser crystallographic software. *J Appl Crystallogr* **40,** 658–674 (2007).
10. Adams, P. D. *et al.* PHENIX: a comprehensive Python-based system for macromolecular structure solution. *Acta Crystallogr. D Biol. Crystallogr.* **66,** 213–221 (2010).
11. Emsley, P. & Cowtan, K. Coot: model-building tools for molecular graphics. *Acta Crystallogr. D Biol. Crystallogr.* **60,** 2126–2132 (2004).
12. Eswar, N. *et al.* Comparative protein structure modeling using MODELLER. *Curr Protoc Protein Sci* **Chapter 2,** Unit 2.9 (2007).
13. Tan, K. P., Nguyen, T. B., Patel, S., Varadarajan, R. & Madhusudhan, M. S. Depth: a web server to compute depth, cavity sizes, detect potential small-molecule ligand-binding cavities and predict the pKa of ionizable residues in proteins. *Nucleic Acids Res.* **41,** W314–21 (2013).
14. Hornak, V. *et al.* Comparison of multiple Amber force fields and development of improved protein backbone parameters. *Proteins* **65,** 712–725 (2006).
15. Jorgensen, W. L., Chandrasekhar, J., Madura, J. D., Impey, R. W. & Klein, M. L. Comparison of simple potential functions for simulating liquid water. *J. Chem. Phys.* **79,** 926–11 (1983).
16. Lippert, R. A. *et al.* A common, avoidable source of error in molecular dynamics integrators. *J. Chem. Phys.* **126,** 046101 (2007).
17. Berendsen, H. J. C., Postma, J. P. M., van Gunsteren, W. F., DiNola, A. & Haak, J. R. Molecular dynamics with coupling to an external bath. *J. Chem. Phys.* **81,** 3684 (1984).
18. Darden, T., York, D. & Pedersen, L. Particle mesh Ewald: An N·log(N) method for Ewald sums in large systems. *J. Chem. Phys.* **98,** 10089 (1993).
19. Case, D. A., Babin, V., Berryman, J., Betz, R. M. & Cai, Q. *Amber 14.* (2014).
20. Bakan, A., Meireles, L. M. & Bahar, I. ProDy: protein dynamics inferred from theory

and experiments. *Bioinformatics* **27,** 1575–1577 (2011).
21. Hunter, J. D. Matplotlib: A 2D Graphics Environment. *Comput. Sci. Eng.* **9,** 90–95 (2007).
22. DeLano, W. L. The PyMOL Molecular Graphics System. *(2002)* (2002).
23. Jenik, M. *et al.* Protein frustratometer: a tool to localize energetic frustration in protein molecules. *Nucleic Acids Res.* **40,** W348–51 (2012).
24. Ferreiro, D. U., Hegler, J. A., Komives, E. A. & Wolynes, P. G. Localizing frustration in native proteins and protein assemblies. *Proc. Natl. Acad. Sci. U.S.A.* **104,** 19819–19824 (2007).
